# Movement patterns and connectivity of gilthead seabream (*Sparus aurata*) in the NW Mediterranean Sea

**DOI:** 10.1186/s40462-025-00619-5

**Published:** 2026-02-23

**Authors:** Davide Thambithurai, Tarek Hattab, David M. P. Jacoby, Philippe Lenfant, Bernat Hereu, Fabien Forget, Olivier Breton, Patrick Bonhomme, Eric Charbonnel, Remi Villeneuve, Alexandre Mignucci, Olivier Derridj, Sylvain Blouet, Jérôme Bourjea

**Affiliations:** 1 UMR MARBEC, IFREMER, Univ Montpellier, CNRS, IRD, Sète, 34200 France; 2 School of Biodiversity, One Health & Veterinary Medicine, University of Glasgow, Graham Kerr Building, Glasgow, G12 8QQ UK; 3 Lancaster Environment Centre, Lancaster University, Lancaster, LA1 4YQ UK; 4 Université de Perpignan Via Domitia, Centre de Formation et de Recherche sur les Environnements Méditerranéens - UMR 5110, 52 Avenue Paul Alduy, Perpignan cedex, 66100 France; 5 Departament de Biologia Evolutiva, Ecologia i Ciències Ambientals, Facultat de Biologia, Institut de Recerca de la Biodiversitat (IRBIO), Universitat de Barcelona, Barcelona, 08028 Spain; 6 Parc National des Calanques, Marseille, 13008 France; 7 Parc Marin de la Côte Bleue, Carry-le-Rouet, 13620 France; 8 Terres Australes et Antarctiques Françaises, Saint Pierre, La Réunion, France; 9 Ville d’Agde, Aire Marine Protégée de la Côte agathoise, Agde, 34300 France

**Keywords:** Acoustic telemetry, *Sparus aurata*, Movement ecology, Spatial networks, Fisheries management, Marine protected areas, Mediterranean Sea

## Abstract

**Background:**

Animal movement underpins critical ecological processes and shapes ecosystem resilience. In marine systems, understanding the spatial ecology and connectivity of exploited species is essential for informing conservation and sustainable fisheries management. Despite their ecological and economic importance, the spatio-temporal movement of gilthead seabream (*Sparus aurata*) in the Mediterranean Sea remains poorly understood.

**Methods:**

We leveraged the largest acoustic telemetry dataset ever collected in the Mediterranean as part of the project CONNECT-MED and RESMED, tracking 222 tagged seabream over three years (2019–2022). Using an array of more than 180 strategically positioned acoustic receivers across the Gulf of Lion in both lagoons and the sea, we analysed over 700,000 detections spanning a longitudinal gradient of 200 km. Using individual-based spatial network analysis, we quantified movement dynamics, space use, and connectivity.

**Results:**

Seabream showed strong seasonal migrations, with wide (> 180 km for some individuals) spatial dispersal during spawning (October–March) and localized movements whilst foraging (April–September). Eastward and southward migration linked lagoon nurseries/foraging areas to offshore spawning areas. The Marseille area (Calanques National Park and Côte Bleue Marine Park) was identified as a major spawning region used by fish across the Gulf of Lion. Movement varied with fish size, with larger fish having more complex and dynamic networks. Autumn saw synchronous lagoon emigration and aggregation at spawning sites, with multi-year site fidelity.

**Conclusions:**

Our findings demonstrate size-dependent movement strategies in gilthead seabream and reveal structured connectivity linking lagoon foraging areas to offshore spawning grounds. The concentration of spawning activity near Marseille identifies a key regional hotspot of ecological and management importance. Incorporating these connectivity patterns, ontogenetic shifts, and spatial behaviours into fisheries management will be essential for sustaining seabream populations across the northwestern Mediterranean.

**Supplementary Information:**

The online version contains supplementary material available at 10.1186/s40462-025-00619-5.

## Introduction

The dispersal and migration of animals is a fundamental process in ecology, with important implications for species management and conservation [1–4]. Throughout their life-histories, animals undertake movement across different spatial scales, environments, and for different reasons. These movement patterns can be broadly categorized into four distinct types: dispersive, sedentary, nomadic, and migratory [5, 6], each with a crucial role in structuring ecosystems and populations [7]. Fish exhibit complex movement patterns, transitioning between movement types throughout ontogeny, or as they relocate between spawning grounds, foraging areas, and overwintering sites. In marine fish populations movement and connectivity can enhance resilience to exploitation [8] and a comprehensive understanding of this nuanced spatial ecology is critical for informing effective conservation strategies, such as the design of marine protected areas (MPAs) [9, 10] and the implementation of targeted management measures, such as temporary fishery closures [11]. Although connectivity can have many definitions, we consider it here the movement of organisms across habitats, ecosystems, at different spatial scales, encompassing both physical migrations and ecological interactions that link different environments and their functions [2]. Importantly, movement can differ as a function of morphological traits such as size (e.g. [12]). Assessing how intra-specific connectivity and movement vary with traits such as size and fish origin remains a crucial hurdle for effectively managing and sustaining fish populations.

Recent advances in animal tracking technologies, combined with new analytical approaches, have greatly enhanced our ability to study the spatial ecology of aquatic species, offering deeper insights into movement patterns, habitat use, and connectivity [13, 14]. Acoustic telemetry, a technique that enables simultaneous tracking of numerous individuals across waterscapes, provides an unprecedented understanding of individual animal movement that can be extrapolated to the population level [11, 15–17]. A particularly effective and novel approach for analysing behaviour from acoustic telemetry data is network analysis [13, 18]. While network theory has been widely applied in animal ecology to study social behaviour [19, 20], its application to spatial ecology – notably to model the movements of animals between habitats and the resulting connectivity – remains less frequent. In this context, vertices in a network can be used to represent discrete locations in space – telemetry stations for instance – whilst edges can represent movement, providing a means to quantify and explore functional connectivity (i.e. the ability of organisms to move and use habitats in a landscape, according to their needs and behaviour [18, 21–25]). Once networks are constructed, network metrics can be extracted to describe important structural properties at multiple scales, allowing the generation of hypotheses about where, how and when animals move [26]. Together, acoustic telemetry and network analysis offer a powerful approach for analysing aquatic animal movements [12, 27, 28].

The Gulf of Lion, the largest continental shelf in the Northwestern Mediterranean Sea, extends from Marseille, France, to Cap de Creus, Spain. It features a network of highly productive estuarine lagoons connected to the sea via canals, which serve as key nursery and foraging habitats for numerous fish species, including the gilthead seabream (*Sparus aurata*)—a species of significant ecological, cultural, and economic value in the region [29]. Although this species is known to occupy coastal areas and marine lagoons, particularly during juvenile stages, detailed knowledge of its spatial ecology remains limited and fragmented [30–33]. Most existing studies, often constrained by small sample sizes and a focus on lagoon entry/exit points, provide only a partial view of its movement patterns [34]. Gilthead seabream are believed to spawn exclusively at sea, with larvae entering lagoons in late winter (February–March) and older juveniles (1+ years) following in early spring (March/April). These fish typically forage in lagoons over summer and return to sea in autumn (October), forming a general summer/winter migration pattern supported by mark-recapture, otolith microchemistry, and telemetry studies [29, 34, 35]. However, key questions remain unanswered, including whether some individuals avoid lagoons entirely, whether different subpopulations exhibit similar movement or spawning behaviours at sea, and the extent to which spawning events are panmictic or stock-specific. Individual variation in migration behaviour based on size, foraging location, and lagoon of origin is poorly understood, as are the precise locations of spawning sites and the distances travelled to reach them. To advance ecological understanding and improve management strategies for gilthead seabream in the Gulf of Lion and the broader Mediterranean, a comprehensive, long-term, and spatially extensive assessment of their movement ecology is needed.

Using data from the CONNECT-MED and RESMED projects—the most extensive acoustic telemetry studies conducted in the Mediterranean to date—we analysed movement patterns and population connectivity of gilthead seabream across the Gulf of Lion. Applying spatial network analysis, we constructed individual movement networks to derive metrics and quantify behavioural variation across temporal scales. Gilthead seabream spawn at sea, and the true natal origin of each fish is unknown. In our study, we use tagging location as a practical proxy for origin, allowing us to compare movement patterns among fish foraging in different lagoons. In this study we address three key questions: (i) how movement varies with fish size, (ii) how space use differs between lagoon- and sea-tagged individuals, and (iii) the spatial and temporal dynamics of seabream during the spawning period in the Gulf of Lion. Our approach provides critical insight into seabream movement ecology and informs spatially explicit conservation and fisheries management strategies.

## Methods

### Study System

**Fig. 1:**
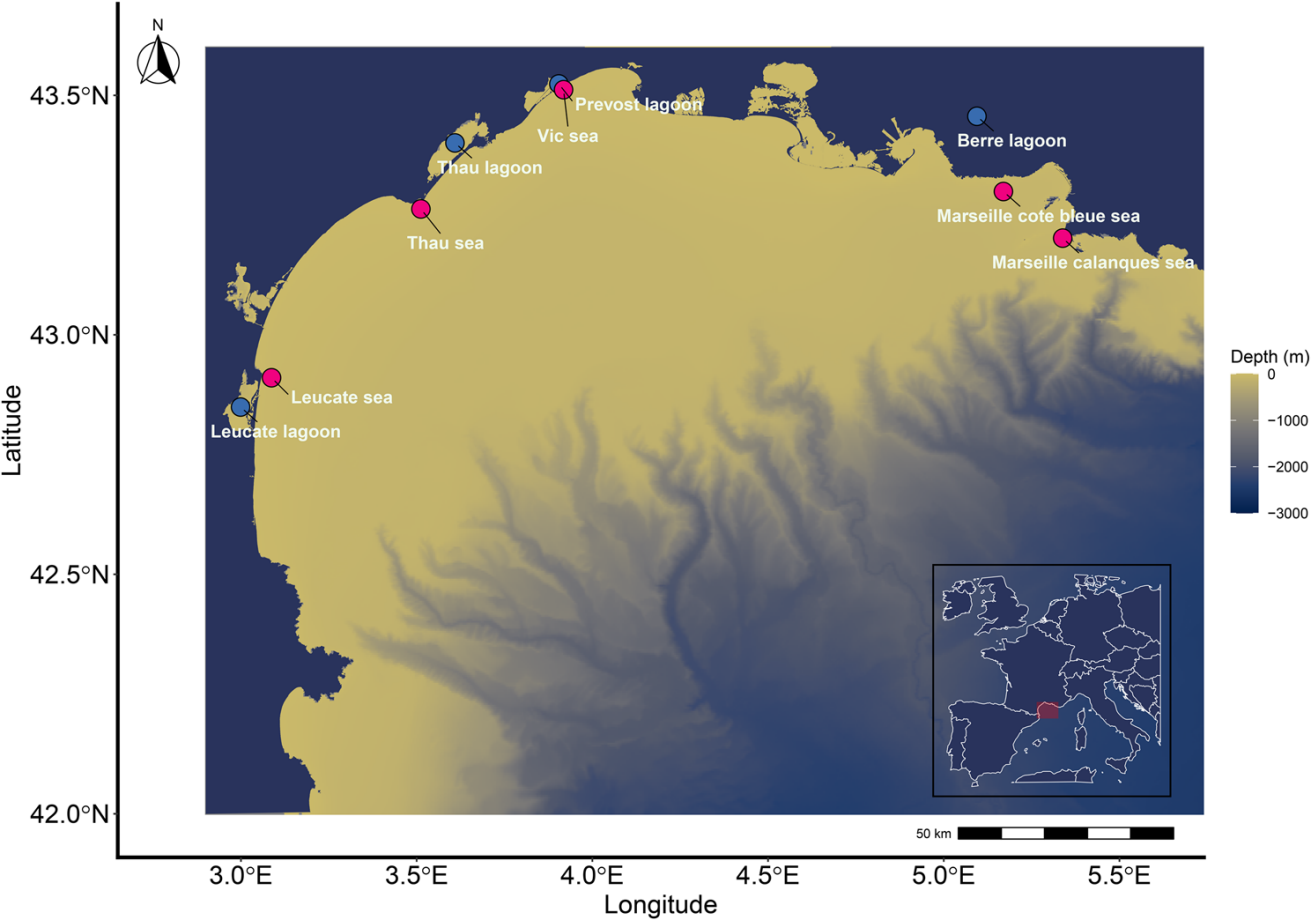
Release locations of tagged fish within the Gulf of Lion study area (French N-Western Mediterranean Sea). Bathymetric map displaying depth contours with tagging sites overlaid. Lagoon habitat sites are indicated by blue points and marine habitat sites by red points. Depth is represented by color gradients from shallow (light) to deep (blue) waters. The inset map shows the regional context of the Gulf of Lion in the NW Mediterranean Sea. The acoustic receiver network configuration is detailed in Fig. S1

The Gulf of Lion is an extended continental shelf measuring approximately 14 × 10^5^ km^2^ in the northwestern Mediterranean, characterised by shallow waters and numerous coastal lagoons (Fig. 1). The lagoons in this region are shallow (1–4 m deep on average), and experience strongly fluctuating environmental conditions with strong weather-driven intra-annual variations in turbidity, temperature, salinity and oxygen [36]. Seabream are found in almost all lagoons that are connected to the sea, although some lagoons are considered more important due to their size and number of fish which use them (e.g. Thau Lagoon, Berre Lagoon and Leucate Lagoon).

### Acoustic network

Regional acoustic tracking in the Gulf of Lion began in 2017; however, the network used in this study became fully operational in April 2019. The full network was a collaborative effort between two joint projects, CONNECTMED (French-led) and RESMED (Spanish-led). Receivers deployed aspart of CONNECTMED were located off the French coast and were more sparsely distributed, becoming active after April 2019. RESMED receivers were deployed closer to Spanish territory and have been active since October 2020. The dataset used in this study is restricted to post-April 2019, with the latest downloads from the array occurring in the summer of 2022. Over the course of the study, 223 VR2W acoustic receivers (Innovasea (formerly Vemco), Halifax, Nova Scotia, Canada) were deployed. However, the number of active stations varied each year due to tampering or technical issues. Some receivers were positioned to monitor ingress and egress points of lagoons (Table S1; Fig. S1), with two receivers placed in close proximity at these locations to ensure high detection probability of fish movement. Mature seabream have been known to display strong easterly movement across the Gulf of Lion during the reproductive season [29], and larvae dispersal modelling has indicated a potential spawning site near Marseille [37]. As a result, a significant number of receivers were also deployed in potential aggregation/spawning sites within and around the Calanques National Park (Table S2). Stations located within canals, lagoons, or inner ports were classified as “lagoon” stations, while those situated in open marine areas were designated as “sea” stations.

### Fish

Seabream (*n* = 222; fork length size range: 182–650 mm; s.d. = 79.7 mm) were collected from sea and lagoon habitats between May 2017 and November 2021 using various fishing methods (Table S3). Tagging occurred across multiple behavioural contexts depending on location and time. Fish were tagged, for example, during foraging within lagoon habitats, at sea during foraging periods, or at sea in spawning areas during spawning periods. These behavioural contexts are based on expert knowledge and published evidence on the species [29, 34, 38]. This species is a protandrous hermaphrodite, with males changing to females at a specific size. In the Gulf of Lion, 50% of males reach sexual maturity at a total length of 240 mm. In this region, gilthead seabream smaller than 250 mm are typically less than one year old and are considered juveniles, whereas individuals larger than this size are considered adults. [39]. Based on published size-at-maturity thresholds [39], fewer than 5% of the individuals tagged in this study were likely to be juveniles.

### Tagging

Following capture, fish were anaesthetised in a 50 L tank containing aerated seawater and 0.025 g L^-1^ benzocaine (Benzocaine ethyl 4-Aminobenzoate). Once anaesthetised, total length (mm) and weight (g) were measured and individuals were placed upside down on a v-shaped tagging cradle. A scalpel incision was made along the central midline anterior to the pelvic girdle, and a coded Innovasea transmitter (V9-1x, delay 130–230 s; V9-2x, delay 60–120 s; V13-1x, delay 80–160 s; estimated tag life 579–1739 days) was implanted into the fish. To close the incision a single suture was typically used, with two sutures used if needed (Monosyn, glyconate monofilament absorbable, needle DS24, thickness 2/0, Braun, Melsungen, Germany). In addition, each fish was dorsally tagged with a spaghetti tag (Hallprint, Hindmarsh Valley, Australia) and tattooed with three dots (Sigma-Aldrich, Missouri, USA) on the pelvic girdle to aid identification in the case of re-capture. Following the tagging procedure fish were given time to recover in a 50 L circular tank filled with aerated seawater until normal swimming was regained (approximately 3–5 min). All fish were released at the same location in which they were captured (Fig. 1). A total of 9 locations ascribed as release sites, four lagoons (Prevost Lagoon, Leucate Lagoon, Thau Lagoon and Berre Lagoon) and five sea sites (Marseille Côte Bleue sea, Marseille calanques sea, Thau sea, Leucate sea, Vic sea).

### Acoustic telemetry data preparation

A series of filtering steps were carried out on the raw data that was extracted from the acoustic receivers, full details of which can be found in Table S4. In addition, detection histories for each of the fish used were visually assessed for extensive periods of non-movement or other erratic behaviour (e.g. static behaviour without movement). An individual was classified as dead if it met any of the following criteria: (i) detections remained fixed at a single station over time in periods where movement is expected (e.g. September to November); (ii) detections persisted long-term but originated from only a few stations with overlapping detection ranges (indicating a deceased fish detected by adjacent receivers); or (iii) movement was extremely limited and consistent with passive drift from tide or currents (i.e., very slow, unidirectional displacement over an extended period, unrelated to seasonal migration) [40]. Each fish was assigned one of two categories depending on the site in which they were captured: lagoon (for fish captured within lagoons) or sea (for fish captured at sea). We also removed fish which displayed no detections after release.

### Spatial network analysis

All data processing and analysis was done in R (version 4.3.2) [41]. Tidyverse was used for data wrangling and mapping, whilst Igraph was used for network analyses [42, 43]. For a full list of packages and reproducible code please consult Script S1, data is also provided in (Supplementary Materials). We constructed network layers for each fish within the dataset using the function *graph_from_data_frame* from igraph. Detections from receiver clusters located at lagoon entrances were pooled into single station arrays (Table S5). This was done to simplify networks at the mouth of lagoons during migration, as in this case we were not interested in this type of fine-scale behaviour. In instances where fish were released and detected at several stations within the same array only one transition within the network was recorded. All network layers used deployed stations as “nodes/vertices”, and movement of fish between stations as “edges/links”. Each layer’s node set was based on the stations available to that individual during the period for which they were tracked, this was a function of either the time between release and recapture, or the time between release and the last detection recorded. We created adjacency matrices for each fish using consecutive detections by the same individual but at different locations, and then used igraph to construct the individual layers. Edge weights were calculated based on the frequency of movements observed between two nodes.

Following the construction of network layers, we estimated a broad set of network metrics to capture complementary aspects of movement and connectivity relevant to our hypotheses (Table S6). Connectivity measures (e.g. edge count, edge density, average path length) describe how extensively individuals moved within the receiver array. Centrality metrics (e.g. degree, betweenness) identify key nodes and pathways, providing insight into the spatial dynamics of spawning migrations and potential management hotspots. Metrics of general space use (sum distance, residency index) quantify overall movement extent and site attachment. This combination ensured that our analysis did not rely on a single property of the networks but instead provided an integrated assessment of space use and connectivity patterns.

## Analysis

### Global movement dynamics and residency

To understand the global movement patterns of gilthead seabream within the Gulf of Lion over time, we aggregated detection data into monthly bins and used this to assess general activity within the acoustic telemetry array. This approach, combined with published evidence [35], and expert knowledge, informed our selection of cutoff periods to define foraging and spawning seasons. Using these time windows, we built two network layers combining data from all years to understand general movement patterns between foraging and spawning periods. To assess the importance of each array in terms of fish residency, we calculated the time fish spent at each location by analysing periods of continuous presence. Continuous presence was defined as more than two detections within a 1.5 hour window, which represents 30 times average delay of tags with a maximum delay of 180 seconds [44]. If fewer than two detections occurred within this period, it was considered the start of a new residency event. Residency events were summed for each fish and array, yielding total residency per array and indicating how long each fish remained at specific locations. We also calculated some distribution metrics from the two network layers to quantitatively test differences between the layers, mean out degree (the mean number of unique departures each node has), mean edge value (the mean number of movements recorded across all locations) and modularity (a reflection of the compartmentalization of the network).

### Community detection

To analyse spatial connectivity within our acoustic telemetry network, we conducted community detection analyses. We first integrated individual fish network layers into a single weighted network encompassing the study duration, disregarding whether movements were from foraging or spawning periods. We applied a log(1+x) transformation to edge weights before running the community detection algorithm. This reduces the dynamic range of the weights, minimizing the dominance of very large values while preserving the relative differences across the network. Community structure was then identified using the Louvain algorithm [45] in igraph setting a default resolution of 1.

### Effect of size and origin on movement

To evaluate how fish length and origin (sea or lagoon) influence space use, we ran a series of Generalized Additive Mixed Models (GAMMs) on data from individual fish spatial networks. Using the gamm() function from the mgcv package [46], we modeled network metrics (Table S6) as response variables, cycling through all metrics while treating fish length and origin as explanatory variables. The small number of juveniles in our fish precluded robust categorical comparisons between juvenile and mature groups. Instead, we modelled fish size as a continuous variable, which allowed us to capture ontogenetic trends in movement while avoiding issues of unbalanced group sizes. Network metrics typically exhibit right-skewed distributions due to the hierarchical nature of ecological networks, where a few highly connected individuals drive connectivity patterns. We applied either a log transformation or a log(1+x) transformation (when zeros were present) to all network metrics to meet model assumptions. To account for potential effects on network metrics, we included time at liberty (i.e. either the period between release and recapture or between release and the estimated end of tag life) as a categorical random factor (short, medium, long, very long). We used the *gam.check()* function from mgcv to ensure sufficient basis dimensions (k) were used in the models.

### Spawning

To identify the peak emigration from lagoons associated with spawning, we firstly subset our data to include detections from only lagoons in which fish were tagged: Prevost Lagoon, Leucate Lagoon, Berre Lagoon and Thau Lagoon. Peak emigration was determined by examining the last detection in a 24 hour period of each fish at any station within its home lagoon (the lagoon where it was tagged) between September and November. The September to November window was based on past tracking work [29], local ecological knowledge (LEK) from artisanal fishermen, as well as the previous seasonal analysis results. Once the last detection of each fish had been identified we grouped all detections for each lagoon into seven-day bins to improve interpretability. Bins with the highest number of individuals were considered as the peak emigration period for each lagoon for that year. To identify peak arrival times at spawning sites among fish foraging locations from various locations, we recorded the first detection at any station considered a potential spawning site (Table S2). This analysis included fish tagged in all locations, and although we did not know the “home lagoon” for sea-tagged fish, we decided to include them to compare their spawning aggregation timings with those tagged in lagoons. Finally, to understand peaks in presence at spawning sites and how each lagoon was represented, we quantified the number of individuals seen over each of the spawning seasons.

## Results

### Acoustic detection data summary

Following initial filtering, 794,169 detections between 15 May 2019 and 4 July 2022 were available from 222 individuals. We recorded data from 136 individuals in 2019, 161 in 2020, 82 in 2021, and 29 in 2022, with several individuals monitored across multiple years. Prior to analysis, and in addition to normal filtering steps, detections were visually checked and individuals displaying behaviours such as extended periods of time without moving were removed (**see Note s1 and acoustic data preparation**).

### Global movement dynamics and residency

**Fig. 2:**
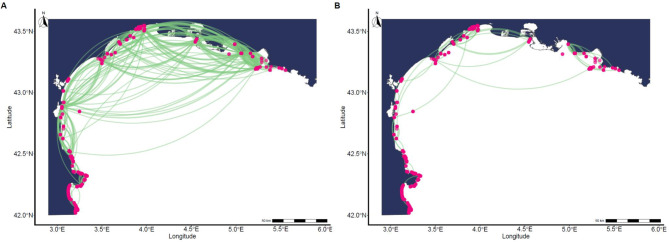
(**A**) regional movement of gilthead seabream during the spawning period – 1 October to 14 April (*n* = 160) – and (**B**) foraging period – 15 April to 30 September (*n* = 35) – for all years (2019–22) between stations in the Gulf of Lion acoustic network. Lines indicate relocations between hydrophone stations and do not represent actual fish trajectories. Note that links (green lines) between release locations and first detections are not shown

**Fig. 3:**
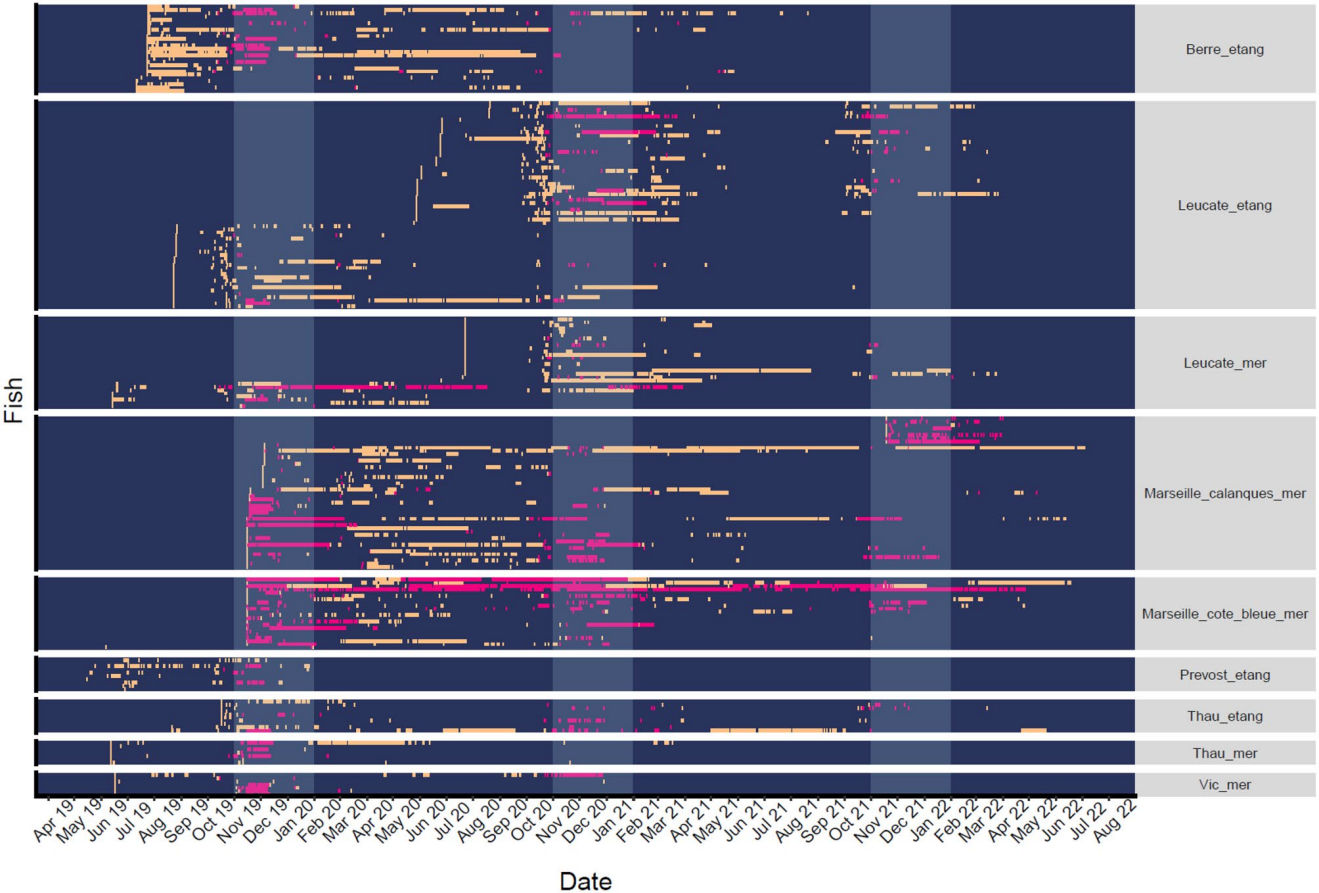
Detections in spawning (pink) and non-spawning (orange) stations for seabream in the Gulf of Lion acoustic network,lines represent individual fish which are grouped by their tagging location shown in panel titles. White shaded areas are indicative of the known peak spawning period (1st of November to 31^st^ of January) for seabream

Fish tagged in different locations exhibited distinct space-use patterns (Figs. S2–S10). Individuals from Prevost, Berre, and Thau Lagoons primarily moved eastward, whereas those from Leucate traveled both eastward and southward toward Spain. Fish tagged at sea in front of Lagoons (Thau, Leucate, Vic) during the foraging season had similar patterns of movements to the fish tagged inside of their nearest lagoons (Figs. S6–S8), however, fish tagged at sea within the spawning areas dispersed more widely across the Gulf of Lion (Fig. S9–S10) and made more intermittent use of lagoons. Detections were highest during the winter months and lowest during the summer (Fig. S11), matching the expected foraging and spawning season of the species. We showed that seabream which forage in lagoons during the summer use marine areas in the winter as a transit zone to reach spawning areas. Based on this, and further published evidence, we decided to construct our global network layers representing two different behaviours (spawning and foraging) with the foraging period defined as April 15^th^ to September 30^th^, and the spawning period as October 1^st^ to April 14^th^. Global network layers revealed that spawning movements were substantially more extensive than foraging movements, particularly in the open sea (Fig. 2), with some individuals migrating across the entire gulf. These differences were reflected in the network metrics for the two layers (spawning vs. foraging): mean out-degree ± SD = 5.42 ± 4.63 vs. 1.84 ± 2.13, mean edge value ± SD = 39.12 ± 339.23 vs. 4 ± 14.63, and modularity was 0.56 vs. 0.67, respectively. The station with the highest mean residency, with fish on average spending more than ~25 days over the lifespan of their tag in the region, was ra_cordiou (see Table S2) located in the expected spawning area within the Calanques National Park. Six of the highest mean residencies were recorded at stations in the same zone, also associated with spawning activity (Fig. S12). Forty-one fish—including individuals tagged at Marseille Côte Bleue Sea and Marseille Calanques Sea—were detected in the spawning region across multiple years: 30 fish visited two subsequent years, and 11, three subsequent years. Several fish also exhibited repeated movements between their home lagoons and the spawning grounds in the Calanques National Park and Côte Bleue Marine Park (Fig. S17). For instance, individuals from Thau Lagoon and Leucate Lagoon were observed migrating back and forward between these areas over multiple years. Some fish, particularly those tagged within the spawning areas, remained within the spawning regions for extended periods of time (e.g. 180  days), although, on average, fish occupied the spawning region less than a week over the study period (Fig. 3).

### Community detection

**Fig. 4:**
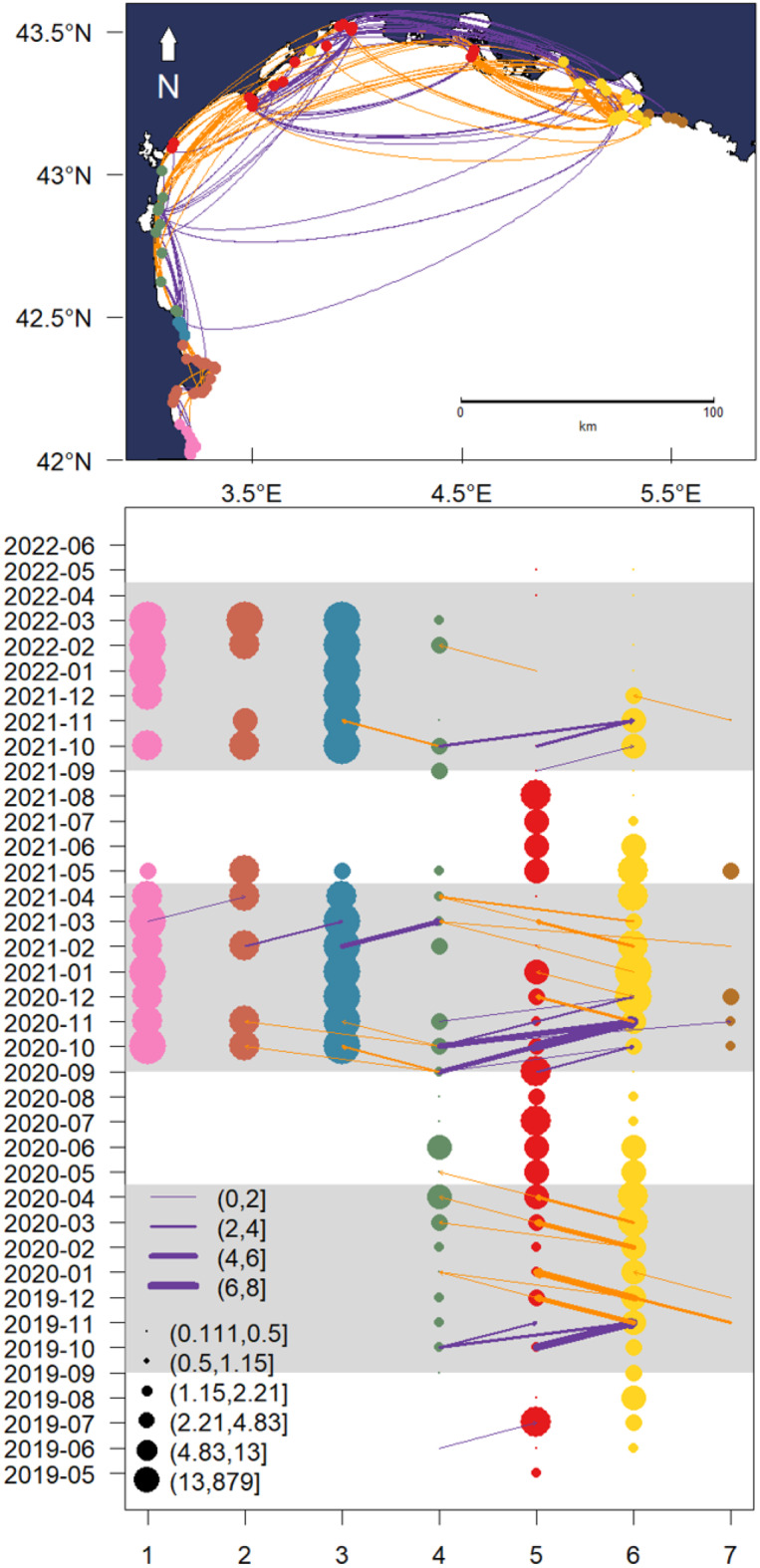
(**a**) Spatial communities on the x-axis of fish-receiver network across the Gulf of Lion as established by the Louvain multi-level modularity optimization algorithm in Igraph with unweighted edges connecting communities shown for east to west movements (purple) and west to east (yellow), colours with community ID are shown in the second panel. (**b**) Movement among communities across the duration of the study, colours are indicative of spatial communities and point size is indicative of monthly average number of individual fish movements within spatial communities. The edge thickness indicates the number of fish that have moved between spatial communities within two consecutive months and point size is indicative of number of fish within the community. Gray and white shaded areas are indicative of the spawning and foraging seasons.

The network community analysis identified seven distinct clusters (Fig. 4). Most communities—i.e. groups of receivers more densely connected with one another by animal movements than with the wider receiver network—were spatially restricted to approximately 40 km. However, Community 5 exhibited a broader distribution, spanning approximately 112 km. A community was assigned to a relatively small number of receivers within the Calanques National Park (Community 7), with a larger community (Community 6) representing the majority of receivers we identified as spawning stations. All communities had at least one connection to another community.

### Effect of size and origin on movement

Network and spatial metrics exhibited consistent relationships with fish size and time at liberty, while effects of origin were not significant after accounting for log-transformed distributions (Table S7, Fig. S15). Connectivity-related measures (mean degree, mean graph strength, edge density) increased significantly with fish length, indicating that larger individuals were connected to more nodes, made stronger connections, and moved through denser portions of the network. For example, the largest individuals of ~550 mm fork length had roughly 2–3 times higher connectivity scores on the log scale than those around 200 mm (Fig. S15). Larger fish also showed longer average path lengths, reflecting broader spatial exploration across the array.

Centrality measures revealed contrasting patterns: max degree centrality increased with size, suggesting that larger fish were linked to more important nodes, whereas max betweenness centrality showed no significant relationship with size or origin once metrics were log-transformed, and instead was mainly influenced by time at liberty.

Metrics of general space use showed mixed patterns. Residency index increased with size—by nearly two log units between 200 and 600 mm—and with time at liberty, consistent with strong site fidelity. In contrast, total distance travelled showed no clear relationship with size, origin, or monitoring duration.

Taken together, these results highlight fish size and monitoring duration as the dominant drivers of spatial network structure, while differences between lagoon- and sea-origin fish were not significant.

### Spawning dynamics

**Fig. 5:**
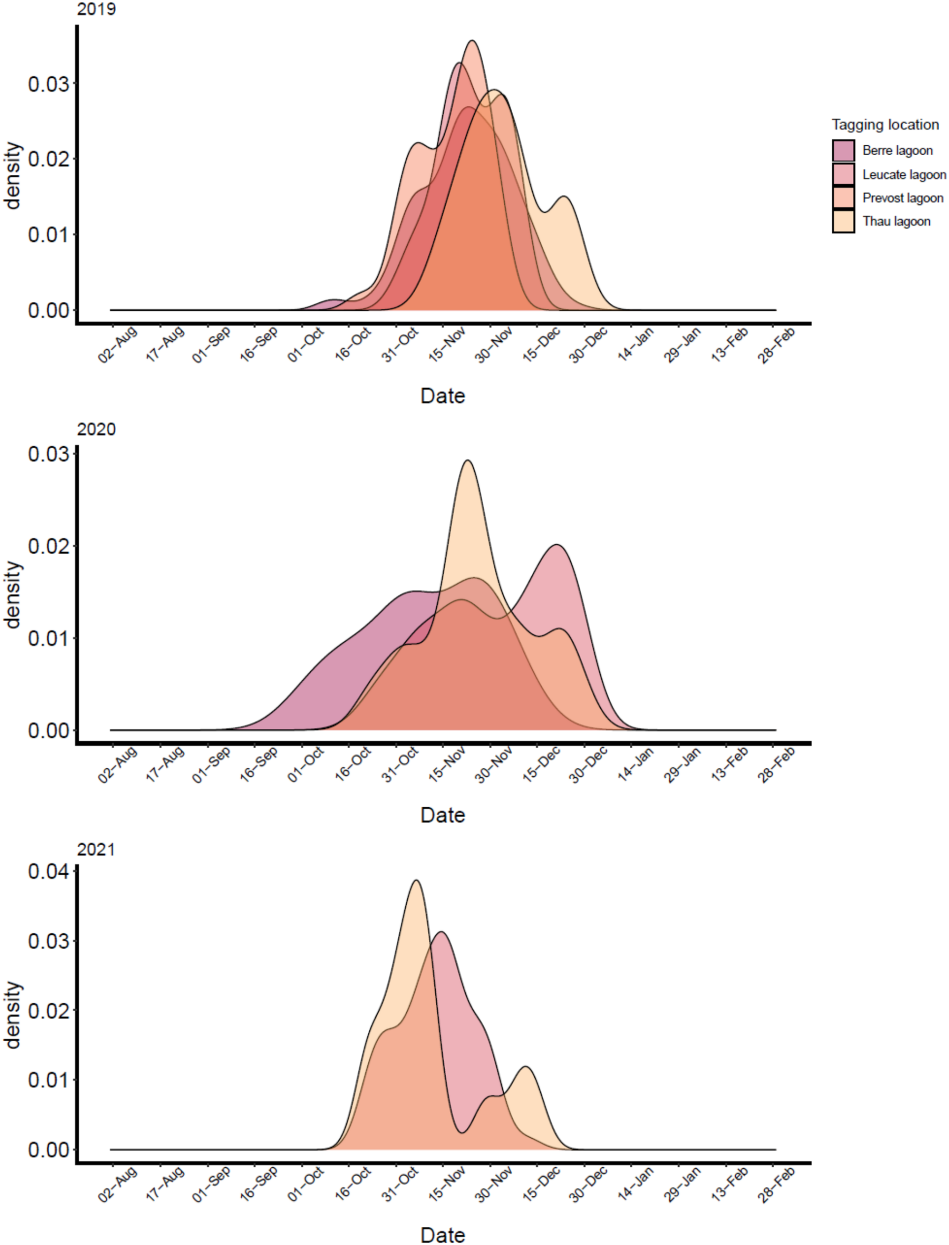
Density functions showing the number of individuals present at the spawning stations over the three spawning seasons as a function of time and tagging location, 2019 (top), 2020 (middle) and 2021 (bottom)

Seaward migration for reproduction generally occurred between October and November across most lagoons and years, with peak activity around mid-October (Fig. S13). Fish from Berre Lagoon, however, showed earlier migration, with some individuals leaving the lagoon as early as September. Exact migration timing varied among lagoons, with consistency among years. Arrival at spawning locations also differed among lagoons. Fish released in Berre Lagoon were the earliest to arrive, except for those tagged in Marseille Côte Bleue sea, which reached the spawning grounds first. (Fig. S14). We found high synchrony in arrival between spawning seasons, and clear evidence that the area in Marseille is used for spawning by fish from across the Gulf of Lion (Figs. 3, 5)

.

## Discussion

### Global results summary

Connectivity in fish populations, especially during spawning, remains a critical yet understudied aspect of marine ecology [47]. In this study, we reveal the movements of gilthead seabream in the Mediterranean, characterized by consistent site fidelity across seasons and high spatial connectivity. A two-phase migratory cycle was identified: a summer foraging phase (April–September) in coastal lagoons with limited and localized movement, and a winter spawning phase (October–March) at sea, marked by extensive oriented long-range movement. Spawning itself was not directly observed, but the repeated presence of ripe females and running males near Marseille, and complementary egg and larval dispersal modelling [37] strongly suggest active spawning in or near Marseille area (the Calanques National Park and the Côte Bleue Marine Park). Our results show that, year after year, fish from across the Gulf of Lion consistently converged in this region, indicating a shared regional spawning site. This spawning site appears to be a key area for the gilthead seabream. Our results suggest that a substantial proportion of the regional spawning biomass is concentrated in this area and could be vulnerable to fishing without effective management. These findings contribute significantly to understanding the spatial ecology and life-cycle of one of the most emblematic Mediterranean fish species and raise important questions for its sustainable management and conservation.

### Movement dynamics and life-history strategies

During the foraging phase, most of the fish tagged in lagoons remained in lagoons (Fig. S16), supporting prior findings from Prévost Lagoon where seabream only exited lagoons under heatwave conditions or during spawning [48]. Juvenile seabream rely heavily on lagoons for feeding in their first year of life, but are thought to frequently continue to use lagoons up to their fourth year of life [34]. Out of 69 individuals tagged during spawning at sea, all adults of considerable size (Fig. S18), 44 (64%) were never observed in lagoons, indicating potential cessation of lagoon use beyond a certain size. A potential alternative, is the existence of a distinct marine “sea stock”, supported by evidence from the North African coast (~800 km away from the Gulf of Lion) showing two morphological seabream phenotypes: one lagoon-associated and another marine-restricted [32]. Movement patterns during the spawning phase were broadly consistent across tagging sites and aligned with earlier studies [29, 31, 49]. Most individuals migrated eastward at the end of the foraging season (Figs. 4 and S2–S10), likely taking advantage of prevailing east-to-west currents [50], which help drift larvae into lagoons from spawning regions to the east, and deeper water near the edge of the shelf. Given the limited swimming capacity of juveniles (~10 cm s^−1^) [51], eastward spawning likely optimizes larval retention to lagoons. Fish tagged in western sites, such as Leucate, exhibited the most complex and variable movements—some traveled south toward Spain, others east toward Marseille—suggesting flexible but regionally coordinated spawning strategies.

### Site fidelity

Site fidelity in animals is considered advantageous as it allows individuals to return to familiar sites to spawn or forage, serving to maximise their fitness [52]. Evidence for return use to previously occupied sites in fishes is particularly strong for anadromous species. In salmonids, natal homing has been extensively documented because individuals can be reliably trapped and monitored in rivers [53, 54], although their marine phase remains less understood due to tracking difficulties [55]. In contrast, less is known about repeated site use in fully marine fishes. A number of species including black seabream (*Spondyliosama cantharus*), Atlantic cod (*Gadus morhua*), and red drum (*Sciaenops ocellatus*) have demonstrated site fidelity to spawning grounds, where individuals were tagged and re-identified across years visiting the same area [56–59]. Only a few studies have tracked movements that span both foraging and spawning grounds, especially in non-pelagic species [60]. Some research also shows marine fish returning to previous capture sites after release elsewhere during foraging [61, 62]. In our study, fish displayed inter-annual site fidelity to both spawning and foraging sites. Many fish returned to the same spawning grounds over consecutive years, and many also showed fidelity to the original foraging sites where they were tagged, and presumably where they first settled. This is remarkable, as during migration these fish are heavily targeted by both recreational and commercial fisheries and must navigate a gauntlet of nets and lines when moving to and from their home lagoons. From an evolutionary standpoint, this suggests that the benefits of undertaking such migrations outweigh the risks. This behaviour was especially strong in fish from Berre Lagoon (Fig. S17), the tagging site closest to the spawning zone, but was also present in fish tagged elsewhere. Given the high fishery related mortality these fish incur [63], it is not surprising that the most represented group at spawning were Berre lagoon fish. These individuals travelled less distance, potentially reducing exposure to fishing while migrating, and maximising cumulative survival to spawning grounds. Of the fish detected during multiple foraging seasons (excluding the tagging year), all but two returned exclusively to their capture lagoon, demonstrating high spatial fidelity. This supports earlier findings in Prévost Lagoon showing fine-scale fidelity to even specific lagoon areas [48].

Previous work has reported limited or no site fidelity in *S. aurata* [29, 31, 34, 48], whereas our results reveal extensive migrations with philopatry to both spawning and foraging sites, aspects that have not been fully addressed in earlier studies. The underlying mechanisms remain unknown but likely involve geomagnetic, olfactory, celestial, social cues [64], possibly changing depending on context. While we didn’t investigate larval homing to natal lagoons, this cannot be ruled out and may involve a random lagoon entry followed by imprinting, as seen in salmonids [55]. This would serve to explain why in the following years fish return to their “home lagoon”. This lagoon fidelity and spatial structuring should be considered in future studies and management strategies.

### Community structure and network metrics

Community structure analysis revealed strong spatial clustering, a pattern commonly observed in marine fish space use [28, 65]. Notably, Berre Lagoon and much of the spawning region formed a single module (community #6), indicating frequent and direct connectivity. Another module (community #7) was centred on the core spawning receivers, suggesting that spawning sites act as distinct network hubs [66]. Movements between communities #5 and #6 around November matched observed spawning migrations from West to East (Fig. 4) [29]. Most seabream traversed the Gulf of Lion via shallow waters ( < 30 m), as shown by sequential longitudinal detections across the acoustic network. This type of coastal movement, both spatially and temporally clustered, makes these fish particularly vulnerable to fishing along their migration routes, something that should be considered in the management of this species [65]. However, larger individuals appeared to take more direct routes, potentially using deeper paths not covered by receivers—supported by a positive relationship between fish size and average path length in GAMM results. Mechanisms proposed to explain this type of ontogenic deepening include natural habitat preferences as a function of size, but also as a response to selective fishing in shallow areas [67, 68].

Our study highlights the value of applying network metrics to quantify individual-level spatial connectivity and movement of marine fishes. By grouping metrics into categories of connectivity, centrality, and general spatial use, we were able to capture complementary aspects of how fish interact with the telemetry array. Using GAMMs to test the effects of size, origin, and time at liberty provided a framework for linking statistical outcomes to ecological processes, allowing us to interpret movement patterns in terms of both individual traits and broader spatial dynamics (Table S7).

Larger individuals consistently exhibited higher connectivity: mean degree, mean graph strength, edge count, and edge density all increased significantly with size, while origin showed no detectable effect. The partial effect plots indicate steep size-related gradients, with larger fish more than doubling their connectivity relative to smaller conspecifics. This suggests a biologically meaningful scaling effect, rather than a statistical one. Time at liberty also had strong positive effects, reflecting the accumulation of contacts and space use with longer monitoring duration. Ecologically, this indicates that larger fish not only engage with more nodes in the array but also move through denser and more integrated portions of the network, thereby broadening their spatial reach during spawning. Such patterns are consistent with the idea that larger individuals often have greater migratory capacity, due to reduced relative locomotion costs for instance [54, 55]. This expanded spatial use by large individuals may enhance opportunities for interaction, mating, conditions for offspring and ultimately recruitment [56, 69, 70].

Centrality metrics quantify the relative influence of locations within a spatial network [57]. Max degree centrality increased with fish size, indicating that larger individuals were more frequently connected to important nodes within the array, which has been found in other species [58, 59]. In contrast, max betweenness centrality showed no significant relationship with size or origin, and was instead primarily driven by time at liberty, reflecting the greater opportunity to accumulate rare transit connections with longer tracking. Origin had no detectable effect on either centrality measure.

Metrics of general spatial use capture the extent and patterns of individual movement. Average path length increased significantly with fish size and time at liberty, indicating that larger individuals travel longer routes, consistent with long-range spawning migrations, or the use of deeper transit zones. In absolute terms, this translates into markedly longer routes for larger individuals, consistent with their broader spatial exploration capacity. Such patterns of longer movement for larger spawning fish have also been noted in other species such as Atlantic cod (*Gadus morhua*) [60]. Residency index also increased with size and tracking duration, reflecting more time spent at specific locations for larger fish. This is potentially linked to spawning behaviour. In contrast, sum distance showed no significant trends, suggesting that total distance moved is less influenced by size or origin. This pattern may be affected by large fish spending more time at sea (out of detection range) during foraging and by the low density of receivers in the sea, which likely leads to underestimation of some movements, leading to a detection bias for lagoon fish [61].

Collectively, these findings demonstrate that individual fish size strongly shapes the structure of the spatial network for *S. aurata*, and that inter-individual tracking duration is an important source of variance. Individual behaviours, such as lagoon fidelity and size-dependent dispersal, scale up to influence population connectivity. Larger fish act as key connectors between lagoons and spawning sites, as well as communities, consistently exhibiting higher connectivity and broader spatial reach, while smaller fish are more spatially restricted. These size-related differences indicate meaningful variation in network position, with larger individuals disproportionately contributing to overall connectivity and spatial cohesion, even if not all centrality measures show strong size-dependence. Future applications of temporal or multilayer network approaches, motif analysis, or flow-based centrality (e.g. [22, 71],) could provide additional insights into seasonal structuring and critical transit nodes, complementing the ecological interpretation of the network descriptors used here. Our initial hypothesis—that at-sea movements of fish tagged in lagoons would differ from those tagged at sea—was not supported, as both groups exhibited similar migration patterns once at sea. The high mobility of large *S. aurata* during the spawning season underscores their important role in maintaining network connectivity in the Gulf of Lion. Incorporating these movement patterns into management and conservation strategies will be critical for ensuring the long-term viability of this species in the region.

### Reproductive ecology

The eastern Gulf of Lion—especially the region near Marseille—was confirmed as a key aggregation ground, with fish from distant lagoons spawning here annually. This highlights its critical role for regional recruitment, at a scale covering the whole Gulf of Lion, especially as larval dispersal modelling links these spawning grounds to multiple coastal lagoons [37]. Spawning site fidelity was clear, with many individuals returning in consecutive years. Arrival timing was broadly synchronized, but fish tagged at Marseille Côte Bleue and Berre Lagoon consistently arrived first, suggesting local foraging or residency. Some fish, especially from Leucate, were never detected at monitored spawning sites, and were seen migrating south towards Spain, despite this they were also detected across multiple foraging seasons, indicating potential use of alternative or additional spawning grounds. One potential site is the Medes Islands where large captures of shoaling seabream in spawning condition are known to occur during autumn and winter (Fig. 4) [72].

Spawning appears to peak mid-November (Fig. 5), but interannual differences in emigration timing suggest environmental cues—like temperature—regulate migration [29, 38, 48, 73]. These cues may shift under climate change, potentially altering phenology, space use and fishery impacts [67, 73, 74]. Compared to many Mediterranean fishes with summer spawning and localized ranges [75], seabream spawn in the autumn and display broad-scale connectivity. The fact that some fish migrate over 180 km to spawn makes them particularly vulnerable to spatially concentrated fishing activity. Our findings showing that fish arrived at spawning grounds in a synchronous manner also raise questions about the social dynamics of migration: do individuals migrate in shoals? Are fission–fusion dynamics at play, enabling group recruitment en route? Do experienced fish lead these migrations [76, 77]? While our results show synchronous emigration and arrival at spawning sites, we have not focused on fission–fusion dynamics or leadership in this study. Future work incorporating high-resolution tracking or social association data could explore these collective behaviours in greater detail [78]. Targeted fishing near Marseille could have wide-ranging effects on population resilience, including downstream impacts on the broader area. This long-range connectivity—potentially spanning multiple countries—must be accounted for in the management of this species. It requires coordinated efforts across institutions, which is often challenging under the current compartmentalised approach to marine management. Conservation strategies and the design of future Marine Protected Areas should encompass the full migratory cycle, including ontogeny, site fidelity, and environmental sensitivity, to support the long-term sustainability of this ecologically and economically important species.

## Conclusion

Our study shows that gilthead seabream in the Gulf of Lion follow a two-phase migratory cycle: a foraging phase, occurring either at sea or in lagoons, and an extensive spawning migration that largely converges annually near Marseille. Larger individuals disproportionately maintained spatial connectivity, while strong site fidelity to both foraging and spawning areas highlighted the stability of these behaviours across years. These results identify the Marseille region as a key spawning hub, where dense aggregations of fish may be particularly vulnerable to fishing. Protecting this area—and integrating long-range connectivity, ontogenetic shifts, and environmental factors into management—will be crucial for sustaining seabream populations from the NW Mediterranean and the fisheries they support.

## Electronic supplementary material

Below is the link to the electronic supplementary material.

Supplementary material 1

Supplementary material 2

Supplementary material 3

## Data Availability

Please see supplementary materials associated to the article, the supplementary material is also available via figshare at 10.6084/m9.figshare.31239709. All acoustic telemetry data is also made available on the European Tracking Network portal (https://www.europeantrackingnetwork.org/en).
