# Supplementary material for: Movement patterns and connectivity of gilthead seabream (Sparus aurata) in the NW Mediterranean Sea
Source: Mov Ecol. 2026 Feb 23;14:10. doi: 10.1186/s40462-025-00619-5 (PMC12927243; doi:10.1186/s40462-025-00619-5)
Supplement: Supplementary file 2 — Supplementary material 2 [file 40462_2025_619_MOESM2_ESM.zip › Raw data.pptx]

## Slide 1
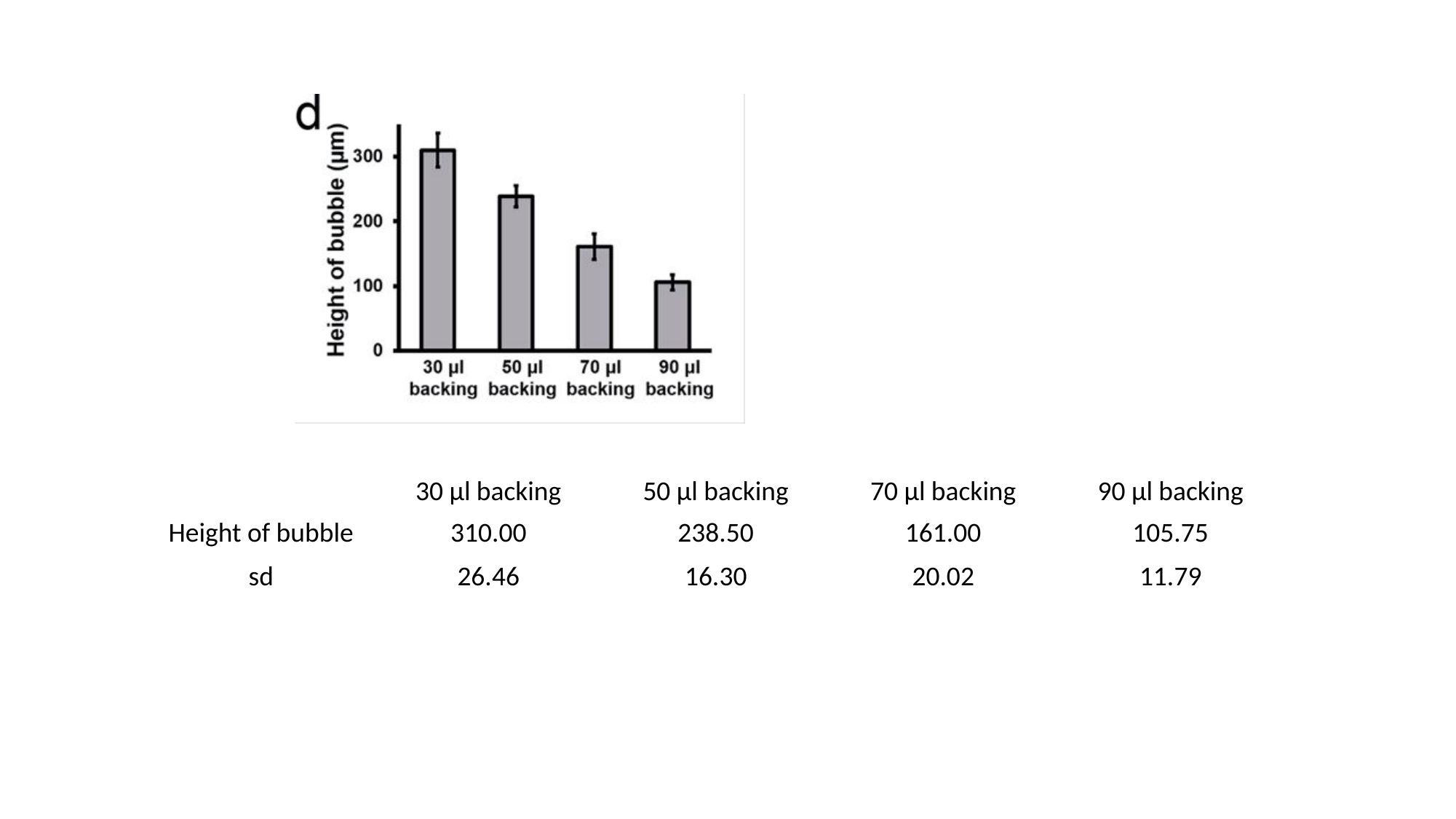

| | 30 µl backing | 50 µl backing | 70 µl backing | 90 µl backing |
| --- | --- | --- | --- | --- |
| Height of bubble | 310.00 | 238.50 | 161.00 | 105.75 |
| sd | 26.46 | 16.30 | 20.02 | 11.79 |

## Slide 2
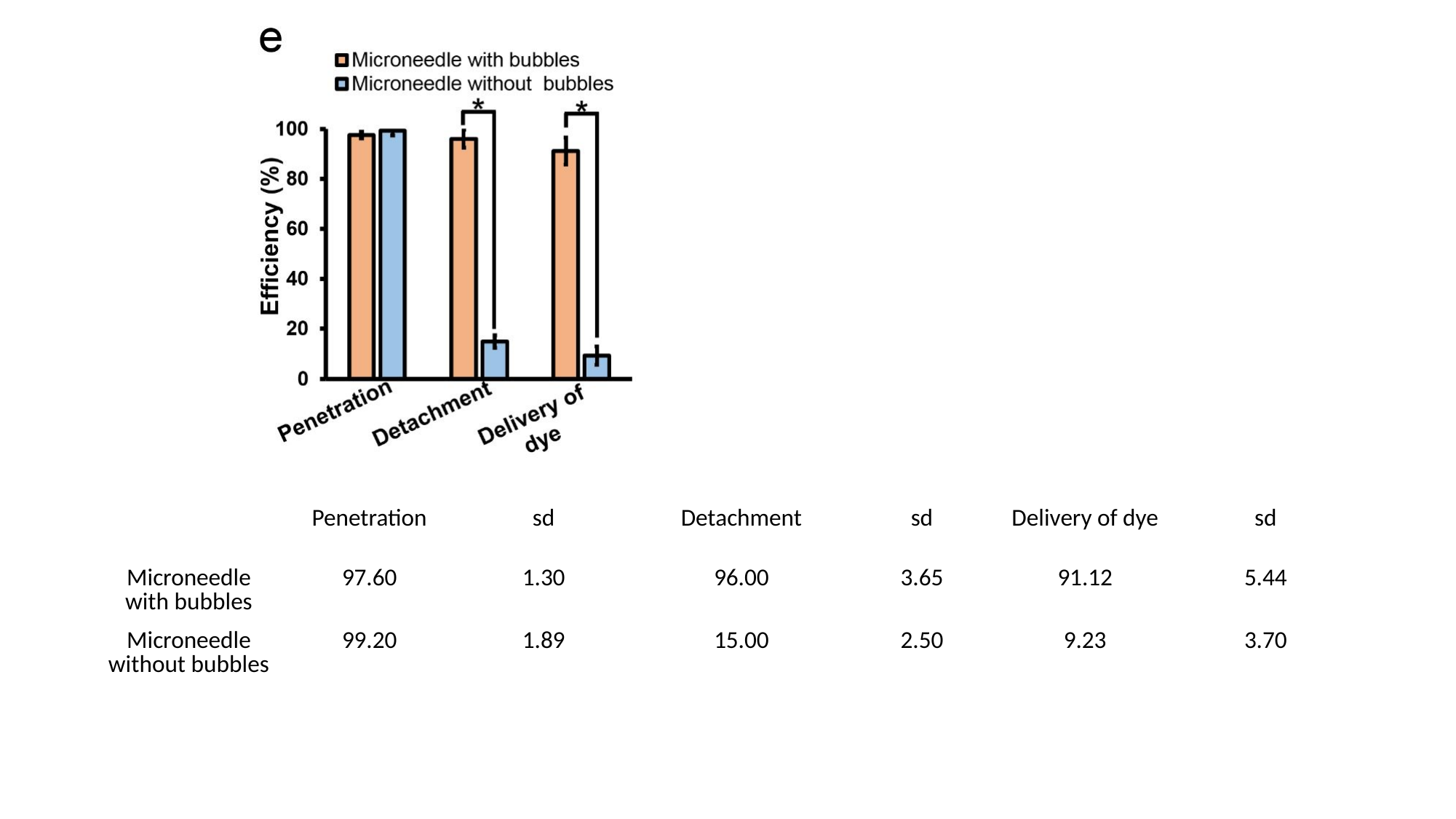

| | Penetration | sd | Detachment | sd | Delivery of dye | sd |
| --- | --- | --- | --- | --- | --- | --- |
| Microneedle with bubbles | 97.60 | 1.30 | 96.00 | 3.65 | 91.12 | 5.44 |
| Microneedle without bubbles | 99.20 | 1.89 | 15.00 | 2.50 | 9.23 | 3.70 |

## Slide 3
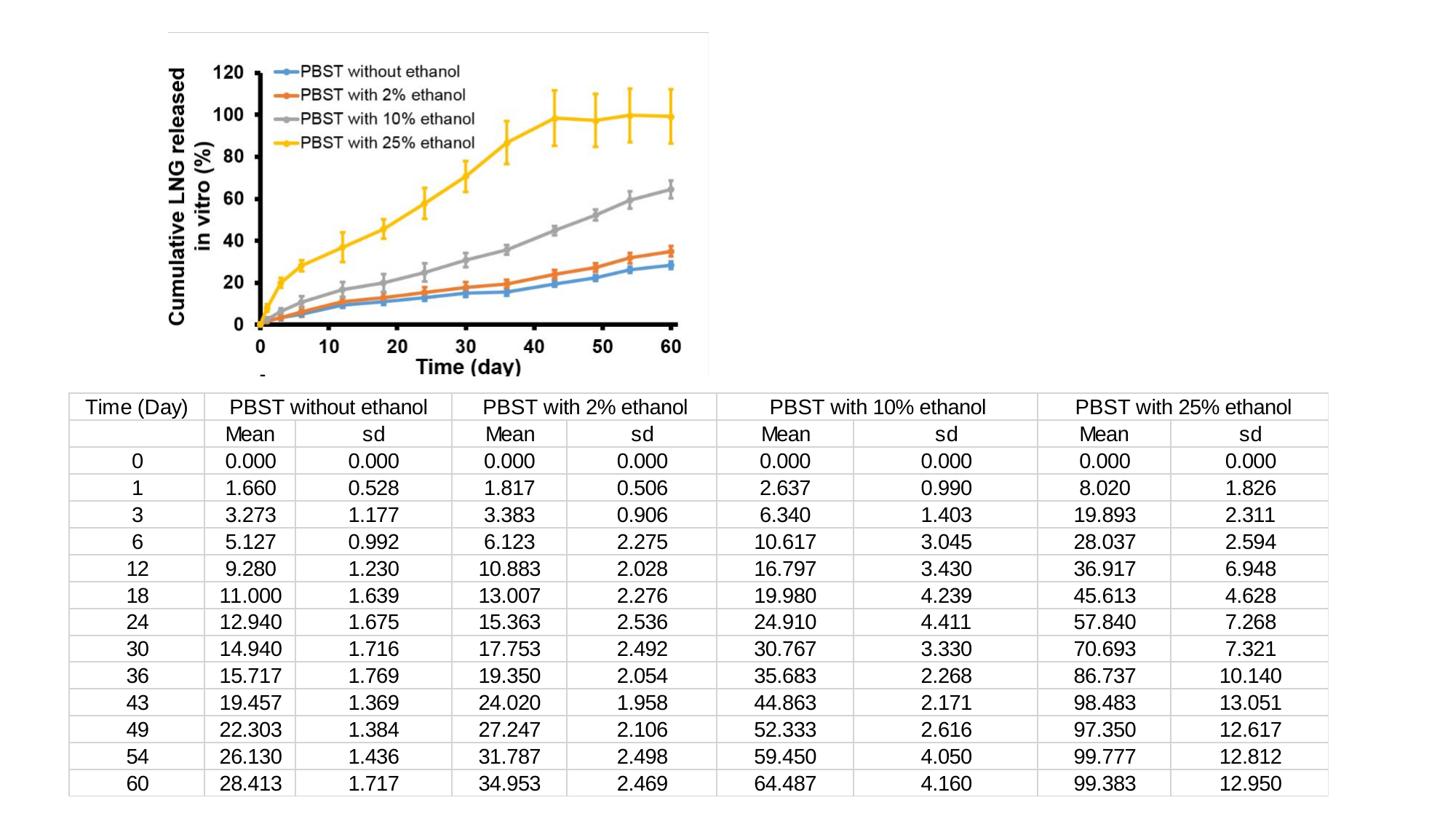

## Slide 4
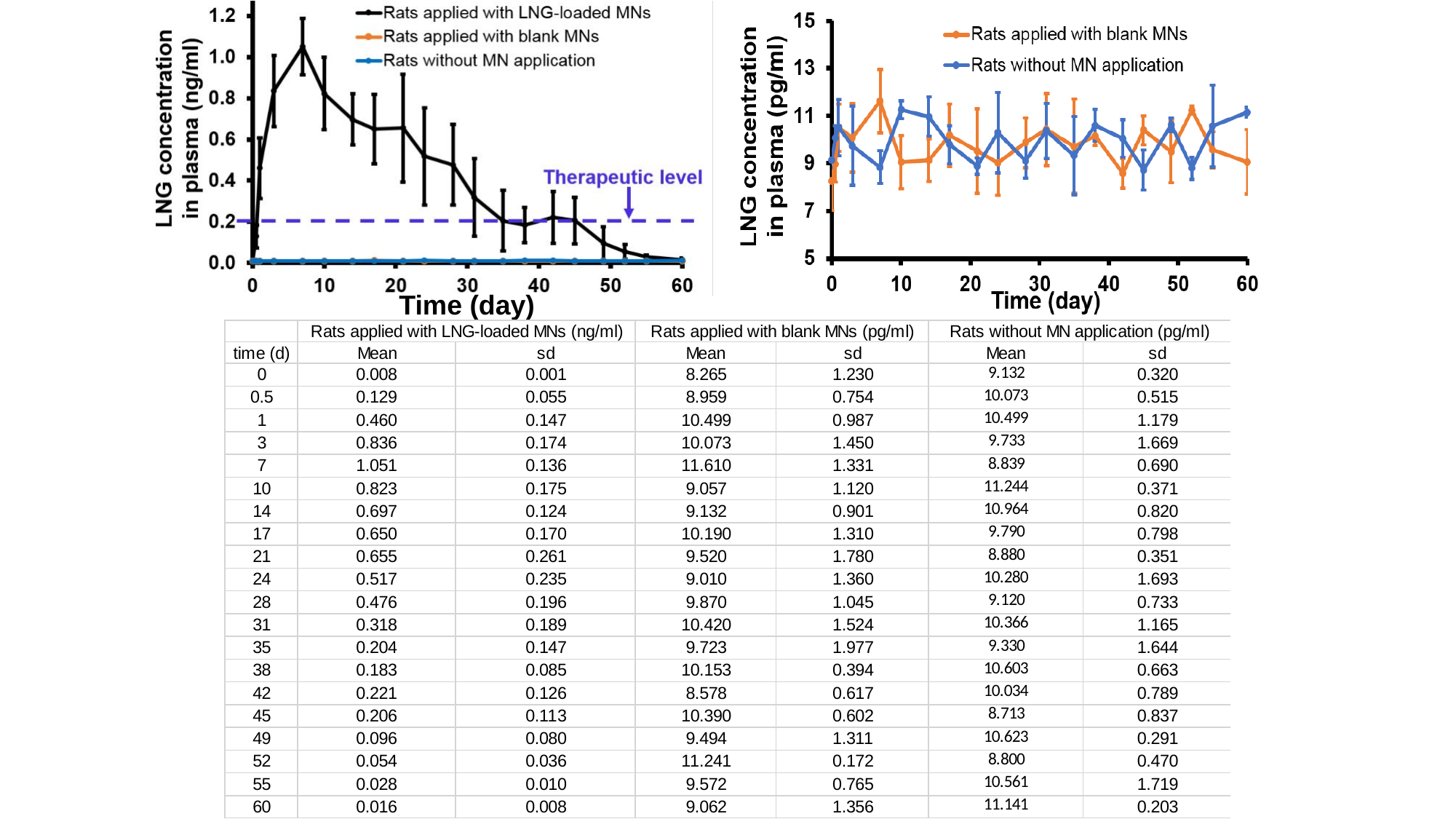

Time (day)

## Slide 5
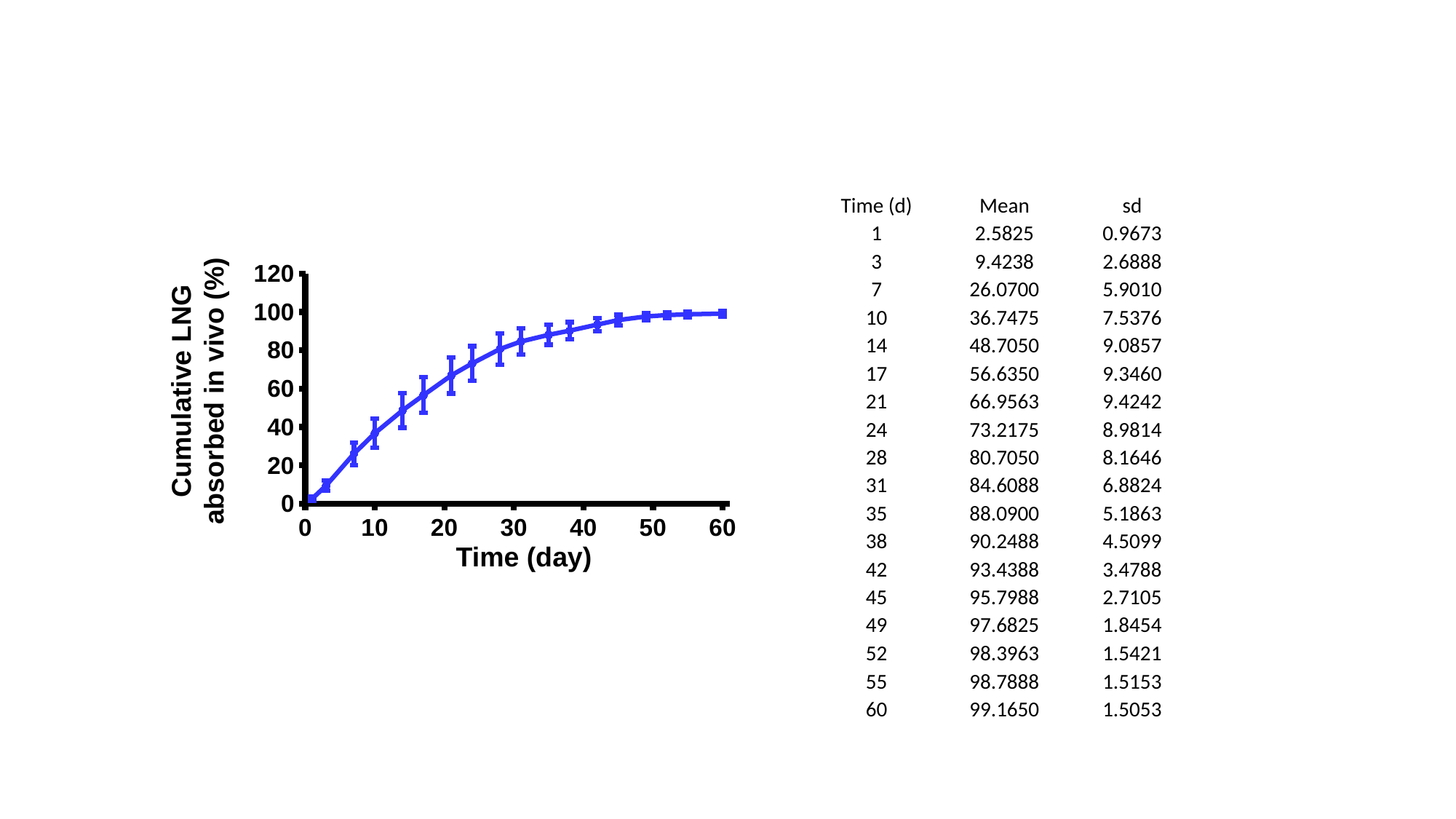

| Time (d) | Mean | sd |
| --- | --- | --- |
| 1 | 2.5825 | 0.9673 |
| 3 | 9.4238 | 2.6888 |
| 7 | 26.0700 | 5.9010 |
| 10 | 36.7475 | 7.5376 |
| 14 | 48.7050 | 9.0857 |
| 17 | 56.6350 | 9.3460 |
| 21 | 66.9563 | 9.4242 |
| 24 | 73.2175 | 8.9814 |
| 28 | 80.7050 | 8.1646 |
| 31 | 84.6088 | 6.8824 |
| 35 | 88.0900 | 5.1863 |
| 38 | 90.2488 | 4.5099 |
| 42 | 93.4388 | 3.4788 |
| 45 | 95.7988 | 2.7105 |
| 49 | 97.6825 | 1.8454 |
| 52 | 98.3963 | 1.5421 |
| 55 | 98.7888 | 1.5153 |
| 60 | 99.1650 | 1.5053 |
### Chart
| Category | Mean |
|---|---|Cumulative LNG absorbed in vivo (%)
Time (day)

## Slide 6
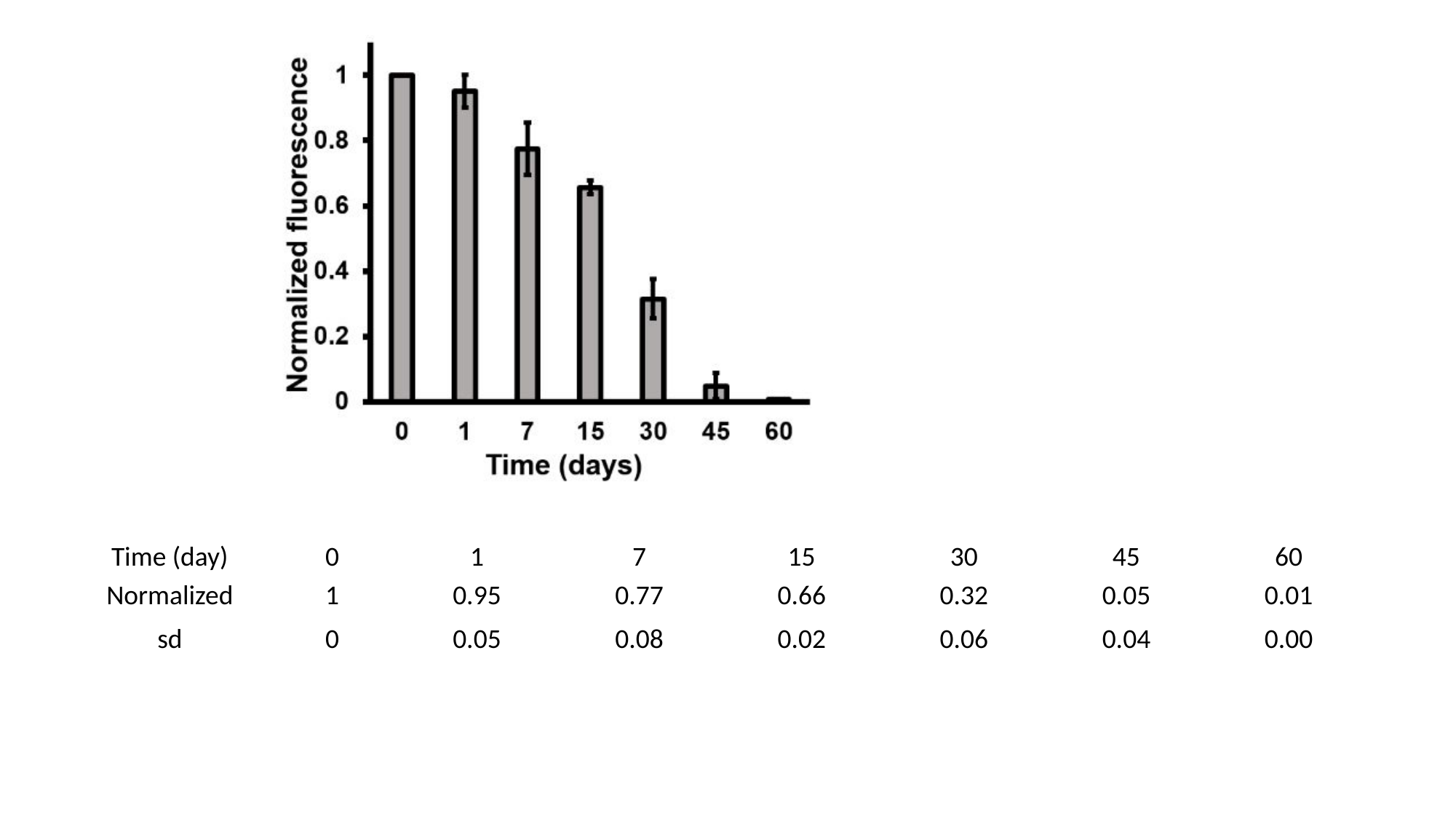

| Time (day) | 0 | 1 | 7 | 15 | 30 | 45 | 60 |
| --- | --- | --- | --- | --- | --- | --- | --- |
| Normalized | 1 | 0.95 | 0.77 | 0.66 | 0.32 | 0.05 | 0.01 |
| sd | 0 | 0.05 | 0.08 | 0.02 | 0.06 | 0.04 | 0.00 |

## Slide 7
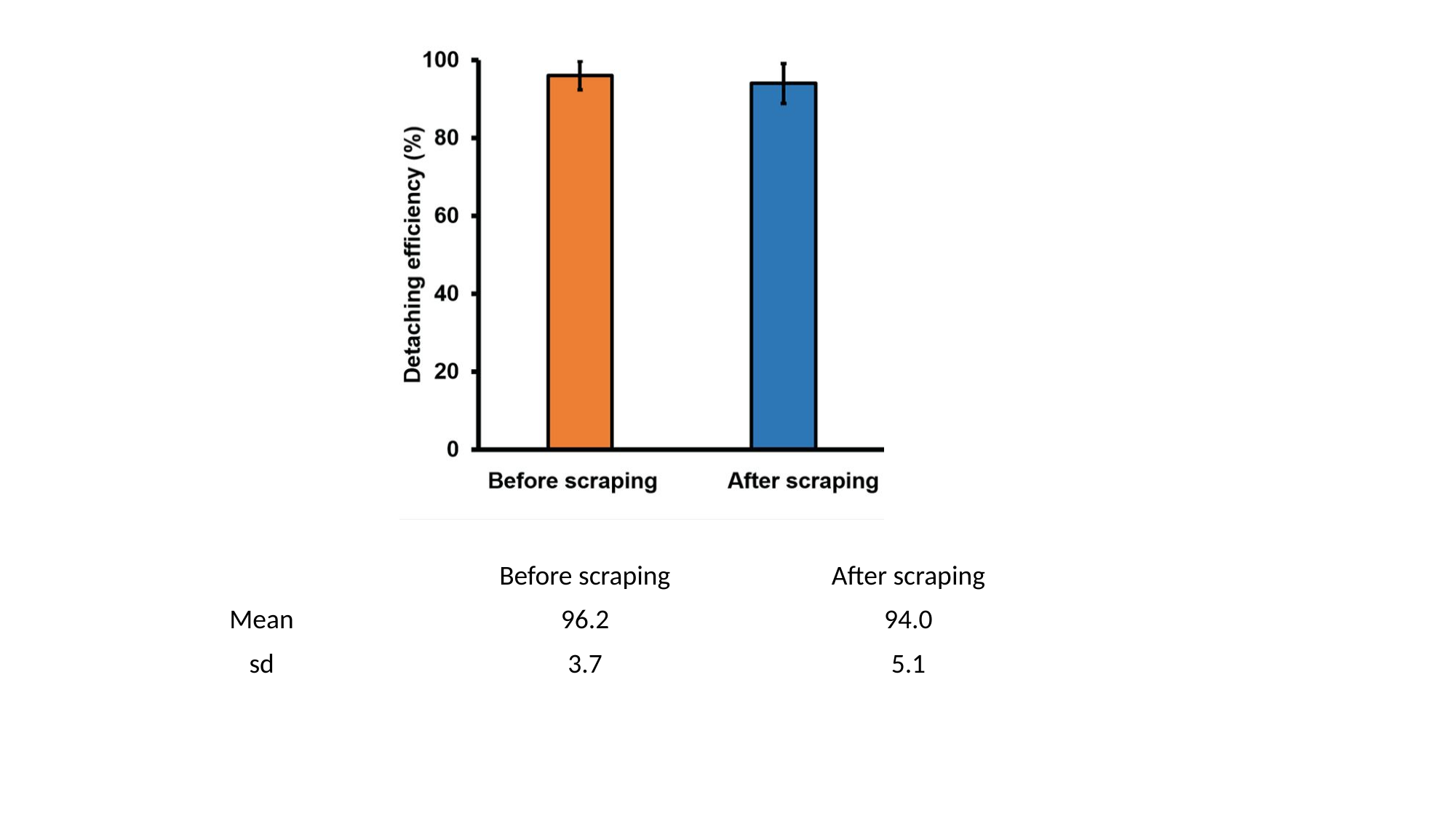

| | Before scraping | After scraping |
| --- | --- | --- |
| Mean | 96.2 | 94.0 |
| sd | 3.7 | 5.1 |

## Slide 8
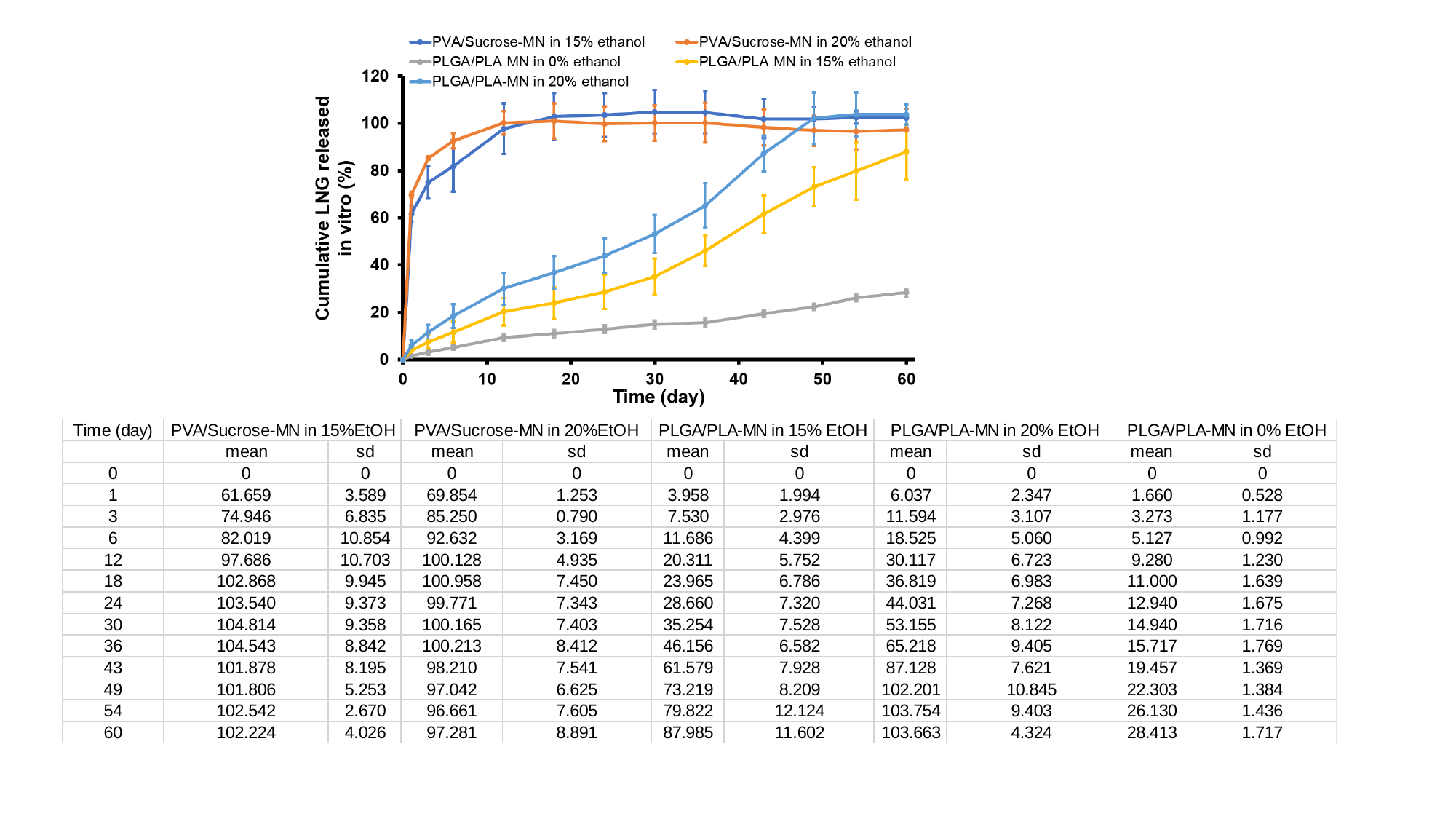

## Slide 9
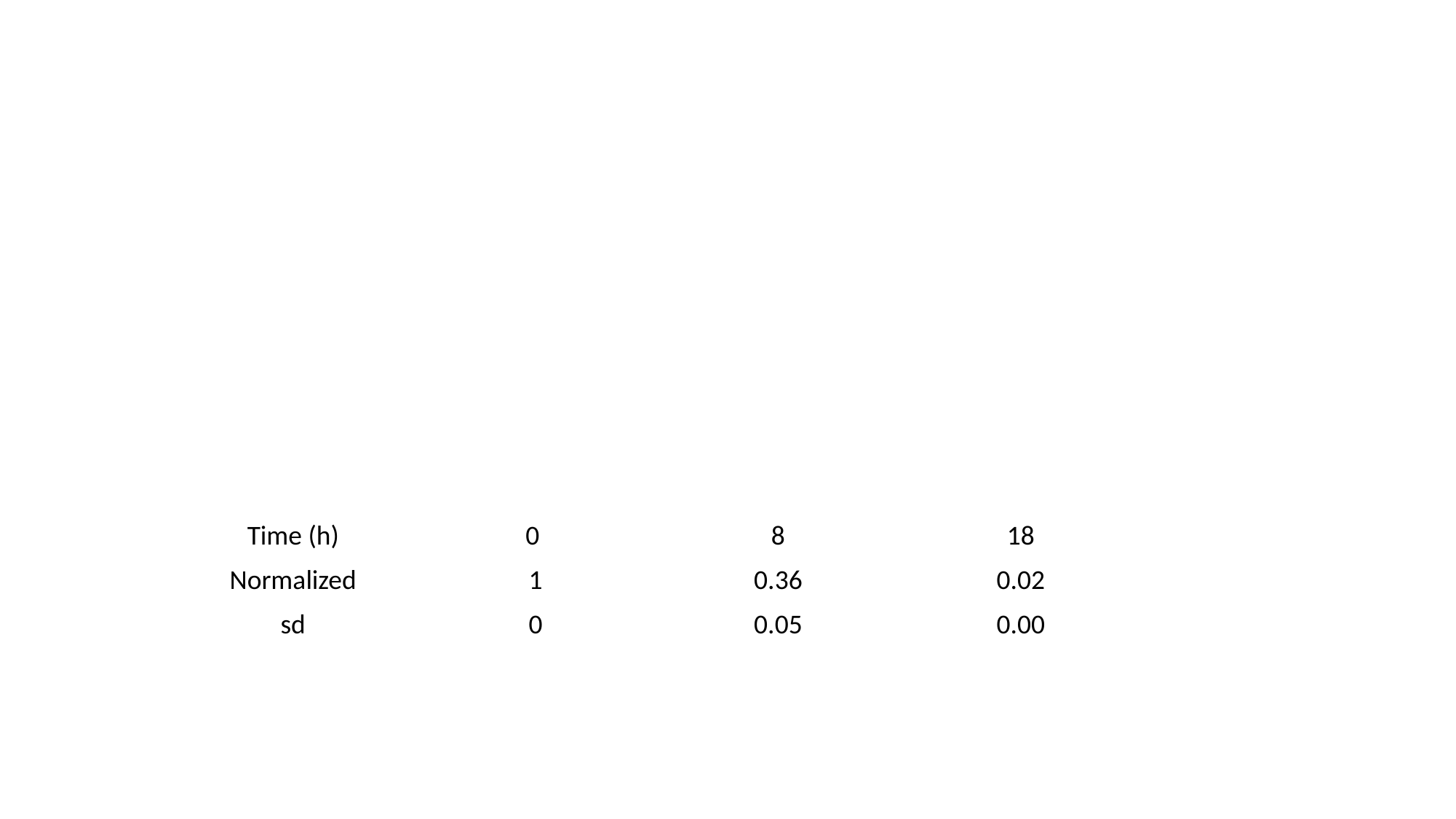

| Time (h) | 0 | 8 | 18 |
| --- | --- | --- | --- |
| Normalized | 1 | 0.36 | 0.02 |
| sd | 0 | 0.05 | 0.00 |

## Slide 10
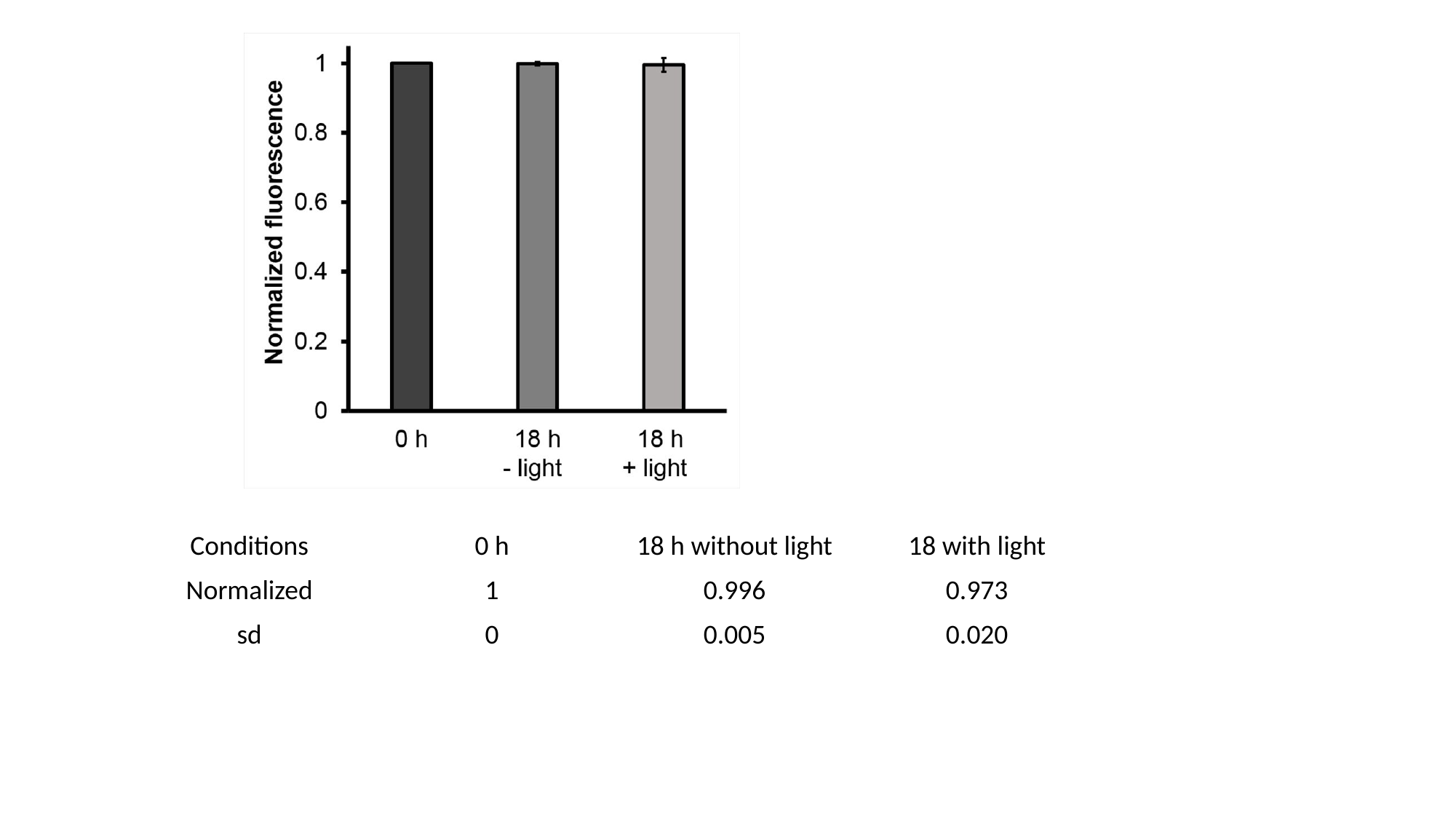

| Conditions | 0 h | 18 h without light | 18 with light |
| --- | --- | --- | --- |
| Normalized | 1 | 0.996 | 0.973 |
| sd | 0 | 0.005 | 0.020 |

## Slide 11
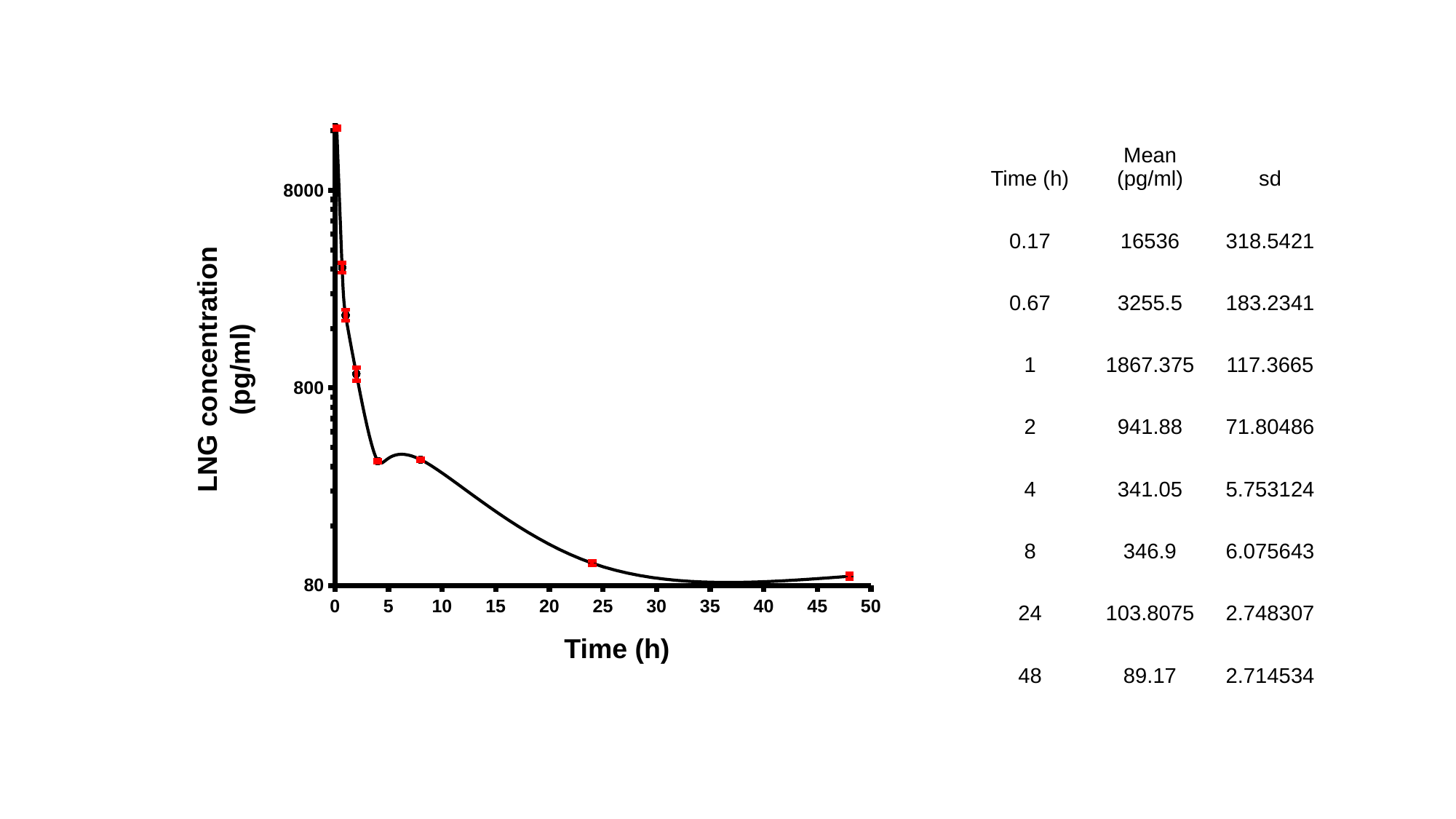

### Chart
| Category | average |
|---|---|| Time (h) | Mean (pg/ml) | sd |
| --- | --- | --- |
| 0.17 | 16536 | 318.5421 |
| 0.67 | 3255.5 | 183.2341 |
| 1 | 1867.375 | 117.3665 |
| 2 | 941.88 | 71.80486 |
| 4 | 341.05 | 5.753124 |
| 8 | 346.9 | 6.075643 |
| 24 | 103.8075 | 2.748307 |
| 48 | 89.17 | 2.714534 |
LNG concentration (pg/ml)
Time (h)
