# Supplementary material for: Movement patterns and connectivity of gilthead seabream (Sparus aurata) in the NW Mediterranean Sea
Source: Mov Ecol. 2026 Feb 23;14:10. doi: 10.1186/s40462-025-00619-5 (PMC12927243; doi:10.1186/s40462-025-00619-5)
Supplement: Supplementary file 3 — Supplementary material 3 [file 40462_2025_619_MOESM3_ESM.docx]

**Supplementary Materials**

**Table S1.** Principal lagoons covered by the array within the Gulf of Lion.

| **Lagoon name** | **Centroid Longitude** | **Centroid Latitude** |
| --- | --- | --- |
| Berre | 5.097659 | 43.456056 |
| Canet | 3.022525 | 42.670129 |
| Grazel | 3.111896 | 43.110477 |
| Gruissan | 3.080416 | 43.112743 |
| Ingril | 3.783374 | 43.442768 |
| Leucate | 2.995777 | 42.848317 |
| Thau | 3.600877 | 43.394365 |
| Vic | 3.831005 | 43.494230 |
| Prevost | 3.902189 | 43.522095 |

**Table S2.** Stations considered as potential spawning sites in the study.

| **Station name** | **Array latitude** | **Array longitude** |
| --- | --- | --- |
| planier_bloc_beton_2 | 43.20915 | 5.268250 |
| planier_bloc_beton_1 | 43.20425 | 5.248350 |
| planier_est | 43.19362 | 5.238869 |
| cote_bleu_couronne_2 | 43.32043 | 5.054367 |
| planier_ouest | 43.20247 | 5.229128 |
| cote_bleu_couronne_1 | 43.32010 | 5.063153 |
| planier_large | 43.19195 | 5.218333 |
| Cote_bleu_corail | 43.32010 | 5.161700 |
| catchoff | 43.29400 | 5.185850 |
| riou | 43.18378 | 5.378783 |
| calanque_devenson | 43.20222 | 5.480833 |
| calanque_soubeyrane | 43.18283 | 5.553333 |
| calanque_cacau | 43.19612 | 5.512500 |
| frioul_tiboulen | 43.27987 | 5.285283 |
| frioul_marte | 43.26152 | 5.287317 |
| ra_prado | 43.26251 | 5.337650 |
| ile_maire | 43.20777 | 5.338867 |
| ra_cordiou | 43.21135 | 5.391933 |

**Table S3.** Details of fish used in the study and associated metadata regarding capture.

| **Capture location** | **Size - Mean ± SD (mm)** | **Capture method** | **Capture location morphology** | **Season tagged** | **No. Individuals tagged** |
| --- | --- | --- | --- | --- | --- |
| Berre lagoon | 287 ± 45 | Barrage net (calen) | Lagoon | Foraging | 27 |
| Leucate lagoon | 304 ± 73 | Long line |  |  | 64 |
| Prevost lagoon | 312 ± 41 | Rod and line |  |  | 10 |
| Thau lagoon | 296 ± 44 | Rod and line |  |  | 2 |
|  |  | Triangle net |  | Spawning | 8 |
| Leucate sea | 312 ± 41 | Long line | Sea | Foraging | 28 |
| Thau sea | 285 ± 20 | Long line |  |  | 7 |
| Vic sea | 298 ± 38 | Long line |  |  | 7 |
| Marseille calanques sea | 385 ± 102 | Rod and line |  | Spawning | 16 |
|  |  | Long line |  |  | 31 |
| Marseille côte bleue sea | 382 ± 50 | Rod and line |  |  | 1 |
|  |  | Long line |  |  | 21 |

**Table S4.** Filtering steps and relative number of data rows at each point. Note that step 1 and 2 include data from other species which are not analysed in this study.

| **Step number** | **What was done** | **Why it was done** | **Number of detections at this step** |
| --- | --- | --- | --- |
| **1** | Load raw data | NA | 11245243 |
| **2** | Removed any tag detection that did not match our transmitter list | To only keep detections from fish that were tagged during the project and remove any others | 3229539 |
| **3** | Removed data from species other than gilthead bream | A number of other species were included in the telemetry study these had to be excluded from this data | 2074565 |
| **4** | Removed any detections from transmitters that were outside the interval for which the transmitter was deployed within a fish | This removes any false signals that for instance were recorded prior to the deployment of tags within a fish (e.g. receiver and transmitter being in proximity outside water) | 1920445 |
| **5** | Joining station data to detections | Station data is needed for downstream analyses | 1487354 |
| **6** | Filter based on transmitter delay. For each transmitter we removed subsequent detections that are below a threshold based on the maximum manufacturer delay. That is to say that if the interval between two subsequent detections is <= the maximum delay of the tag the second detection is removed. This continues until the sum of time between the first detection and all subsequent detections exceeds the max_delay (varies for different tags) | In some cases transmitters can ping simultaneously between two stations that are in close proximity. This filter serves to subset such artifacts from the data. | 865464 |
| **7** | Adding release rows to the data | These are needed for a series of downstream analyses | 865856 |
| **8** | Removed single detections | We decided to remove single detections as we could not verify the validity of these | 865514 |
| **9** | Removed either full or partial detection histories of individual fish | Following an expert review of combined data sources data of some fish was identified as erroneous and removed (see Note S1) | 794349 |
| **10** | Removed release rows representing fish that were never seen following release | Step seven added release rows for all fish in the study, a proportion of these were never seen and were removed from further analyses | ^*^794180 |

* Note that from these rows preceding the 1st of April 2019 were also subtracted in analyses.

**Table S5.** Array name and associated coordinates given to stations with multiple receivers, typically these were present within canals connecting lagoons to the sea. Note that etang means lagoon.

| **Station name** | **Array name** | **Array latitude** | **Array longitude** |
| --- | --- | --- | --- |
| Barcares_barrage | Barcares | 42.79748 | 3.03932 |
| Barcares_mer |  |  |  |
| Berre_etang_1 | Berre | 43.39512 | 4.98459 |
| Berre_etang_2 |  |  |  |
| Berre_mer |  |  |  |
| Canet_etang | Canet | 42.65702 | 3.03493 |
| Canet_mer |  |  |  |
| Carnon_mer | Carnon | 43.54239 | 3.97475 |
| Leucate_conch | Conch | 42.88469 | 3.05287 |
| Leucate_tonton |  |  |  |
| Frontignan_etang | Frontignan | 43.43172 | 3.77727 |
| Frontignan_mer |  |  |  |
| Gruissan_etang_mur | Gruissan_1 | 43.11099 | 3.12666 |
| Gruissan_etang_ponton |  |  |  |
| Gruissan_mer_bloc | Gruissan_2 | 43.09465 | 3.11395 |
| Gruissan_pilone |  |  |  |
| Leucate_barrage | Leucate | 42.87299 | 3.05163 |
| Leucate_ponton_isole |  |  |  |
| Marseillan_etang | Marseillan | 43.31765 | 3.55933 |
| Marseillan_mer |  |  |  |
| Port_restaurant | Palavas | 43.52577 | 3.93471 |
| Port_la_nouvelle_etang | Port_la_nouvelle | 43.01393 | 3.06717 |
| Port_la_nouvelle_mer |  |  |  |
| Prevost_etang | Prevost | 43.51849 | 3.9148 |
| Prevost_mer |  |  |  |
| Sete_mer | Sete | 43.39647 | 3.70479 |
| Sete-etang |  |  |  |

**Table S6.** Network metrics calculated during the study. Vertex, Edge and Fish metrics were used in GAMMs, whilst layer metrics were used in data summaries.

| **Metric name** | **Transformation used** | **On what is the calculation based?** | **Definition** | **Relevance** |
| --- | --- | --- | --- | --- |
| Mean_degree | log() | Vertices | Average number of edges an individual uses | Helps to determine how exploratory an individual is |
| Mean_graph_strength | log() | Vertices | Average edge weight across all nodes | A high mean_graph_strength would indicate a more connected network with more movement |
| Max_degree_centrality | log() | Vertices | Location that has the highest number of direct connections to other stations | Shows whether fish are relying on hub locations (high value) or whether their movement is more dispersed (lower value) |
| Max_betweeness_centrality | log(x+1) | Vertices | The highest betweenness centrality score representing the maximum number of shortests paths between any two stations that pass through a single station | Shows how centralized a fish’s movement on key location is |
| Edge_count | log() | Edges | The total number of unique movement paths an individual has made | Shows global spatial complexity or exploration |
| Edge_density | log() | Edges | The proportion of edges present out of a total number of edges possible in a given network | Captures the efficiency of space use and utilisation of space, a higher density shows that the fish is using more of the space available |
| Average_path_length | log() | Edges | The average shortest path between two nodes in the graph | Yields useful information on the efficiency of movement within the network, or how complicated a route is taken to link nodes |
| Sum_distance | log() | Fish | The sum of distance in km that the fish undertook accounting for all edges across the detection history | Difference in sum_distance between individuals indicate that some fish are undertaking greater movement in terms of total distance covered over detection history, this does not equate to more movement as some fish may undertake many small movements adding to a higher sum_distance |
| RI_index | log() | Fish | Days at liberty (date of release and date last seen/captured) divided by number of days recorded in array | A higher residency index would be indicative of a fish that is more static as more activity would mean a higher number of days no seen in array. |
| Mean out-degree^+^ | Not transformed | Layer | Average number of outgoing connections per vertex (station) | Indicates how widely fish move from each location; higher values suggest more dispersive movement, lower values indicate more localized movement |
| Mean edge value^+^ | Not transformed | Layer | Average weight of edges between vertices | Reflects the intensity or frequency of movement between locations; higher values indicate more repeated use of specific pathways |
| Modularity^+^ | Not transformed | Layer | Degree to which the network is divided into modules or clusters | Measures network compartmentalization; higher values indicate stronger clustering of movement within subsets of locations, lower values suggest more uniform connectivity |

^+^Global network metrics used for general summaries and not GAMMs

**Table S7.** Summary of GAMM results for network metrics. Effects of fish size (long_f), origin (sea vs lagoon), and time at liberty on spatial network metrics. Symbols indicate the direction of significant effects: “+” positive, “–” negative, “ns” not significant. P-values are shown in parentheses. The “Interpretation” column summarizes the ecological meaning of each effect.

| **Model** | **Metric** | **Size (Fork Length)** | **Origin (Sea vs Lagoon)** | **Time at liberty** | **Interpretation** |
| --- | --- | --- | --- | --- | --- |
| log(mean_degree) ~ s(long_f) + cap_li_morphology + s(cat_time_at_liberty, bs = "re") | Mean degree | + (p=0.024) | ns (p=0.246) | + (p<0.001) | Larger fish connect more nodes; lagoon vs sea origin had no effect; longer tracking increases degree. |
| log(mean_graph_strength) ~ s(long_f) + cap_li_morphology + s(cat_time_at_liberty,  bs = "re") | Mean graph strength | + (p<0.001) | ns (p=0.131) | + (p<0.001) | Larger fish make stronger connections; origin had no effect; longer tracking increases strength. |
| log(max_degree_centrality) ~ s(long_f) + cap_li_morphology + s(cat_time_at_liberty,  bs = "re") | Max degree centrality | + (p=0.011) | ns (p=0.594) | + (p<0.001) | Larger fish connect to more important nodes; no effect of origin; time at liberty increases centrality. |
| log(max_betweenness_centrality) ~ s(long_f) + cap_li_morphology +  s(cat_time_at_liberty, bs = "re") | Max betweenness centrality | ns (p=0.131) | ns (p=0.115) | + (p<0.001) | No clear size or origin effects; only longer tracking increases max betweenness centrality. |
| log(edge_count) ~ s(long_f) + cap_li_morphology + s(cat_time_at_liberty,  bs = "re") | Edge count | marginal (p=0.073) | ns (p=0.291) | + (p<0.001) | Weak, non-significant trend for larger fish using more edges; no effect of origin; time at liberty increases edge count strongly. |
| log(edge_density) ~ s(long_f) + cap_li_morphology + s(cat_time_at_liberty,  bs = "re") | Edge density | + (p=0.017) | ns (p=0.231) | + (p<0.001) | Larger fish move through denser networks; no effect of origin; longer tracking increases density. |
| log(av_path_length) ~ s(long_f) + cap_li_morphology + s(cat_time_at_liberty,  bs = "re") | Average path length | + (p<0.001) | ns (p=0.265) | + (p<0.001) | Larger fish travel longer routes; time at liberty increases path length. |
| log(sum_distance) ~ s(long_f) + cap_li_morphology + s(cat_time_at_liberty,  bs = "re") | Sum distance | ns (p=0.487) | ns (p=0.792) | ns (p=0.127) | No clear effects of size, origin, or time at liberty. |
| log(RI_index) ~ s(long_f) + cap_li_morphology + s(cat_time_at_liberty,  bs = "re") | Residency index | + (p<0.001) | ns (p=0.456) | + (p=0.004) | Larger fish show higher site fidelity; residency also increases with longer tracking. |


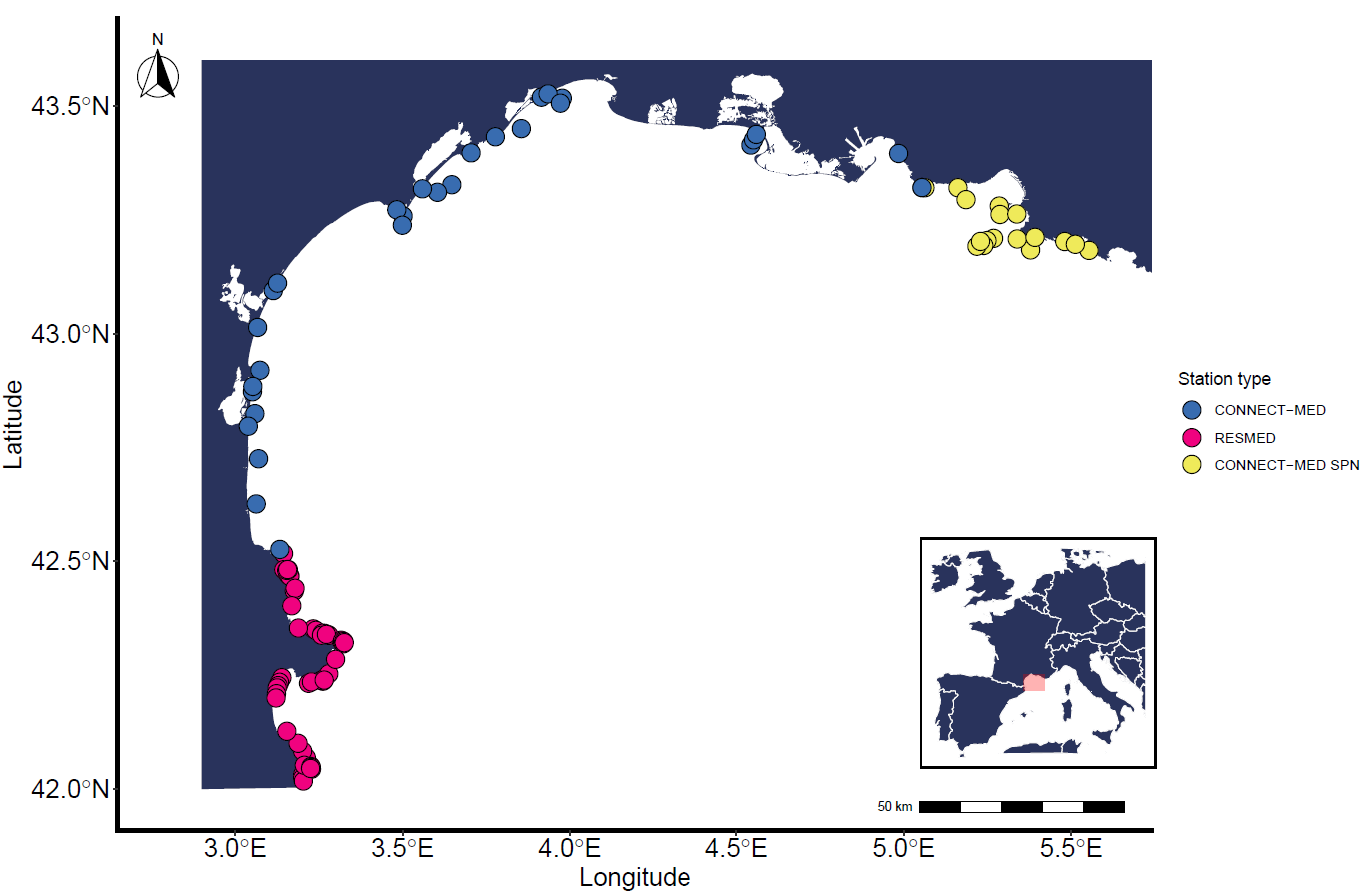


**Figure S1.** Location of acoustic receivers deployed over the duration of the study within the Gulf of Lion, with location of Gulf of Lion in the Mediterranean (inset). Note that the map has been simplified to only show arrays in instances where multiple stations were joint for analysis purposes. RESMED (pink), CONNECT-MED (blue) and CONNECT-MED considered spawning stations (yellow) are shown.


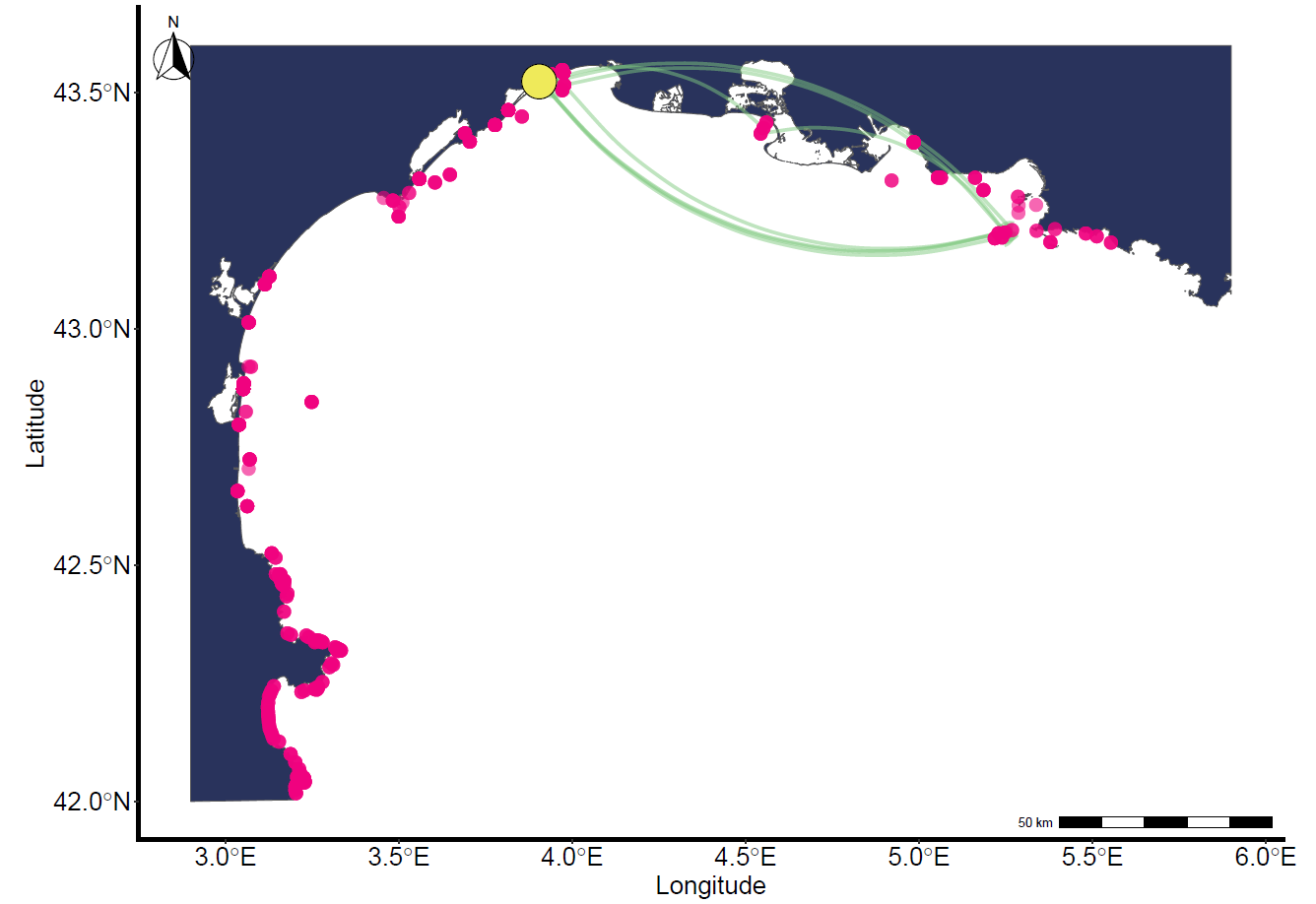


**Figure S2.** Aggregated regional spatial network from fish tagged in Prevost lagoon over the entire study period. Tagging location is shown in yellow, and stations are shown in pink.


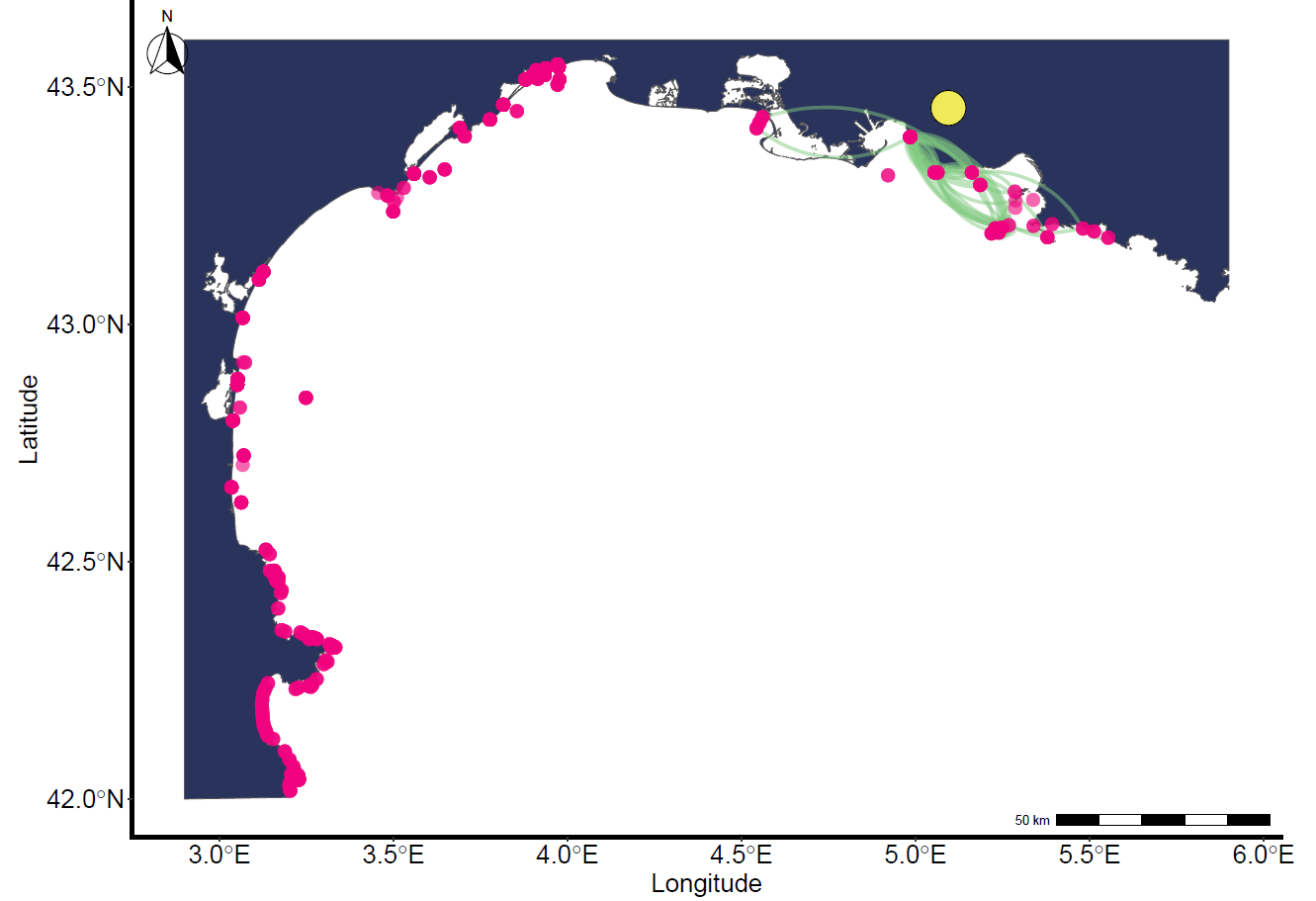


**Figure S3.** Aggregated regional spatial network from fish tagged in Berre lagoon over the whole study period. Tagging location is shown in yellow, and stations are shown in pink.


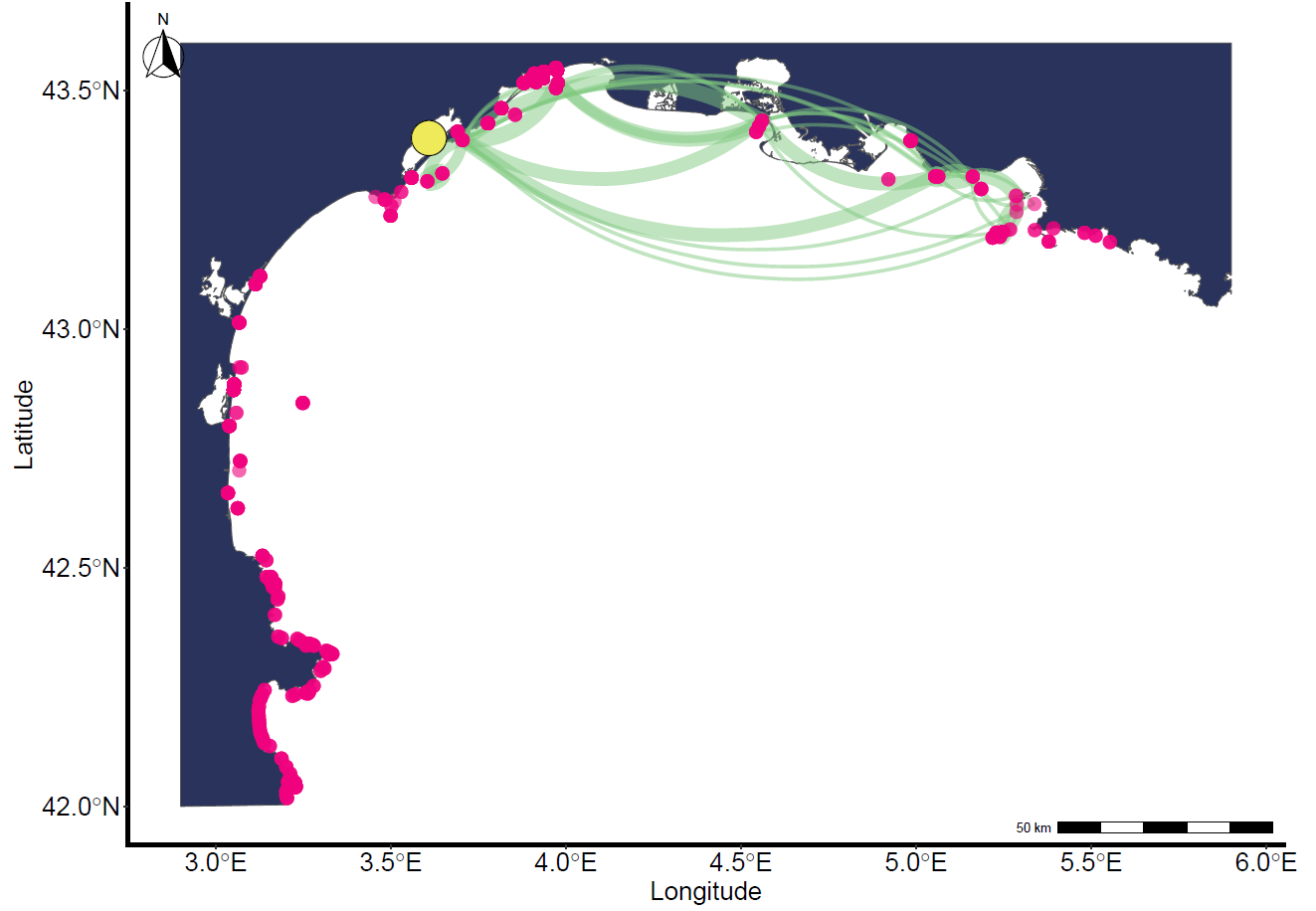


**Figure S4.** Aggregated regional spatial network from fish tagged in Thau lagoon over the whole study period. Tagging location is shown in yellow, and stations are shown in pink.


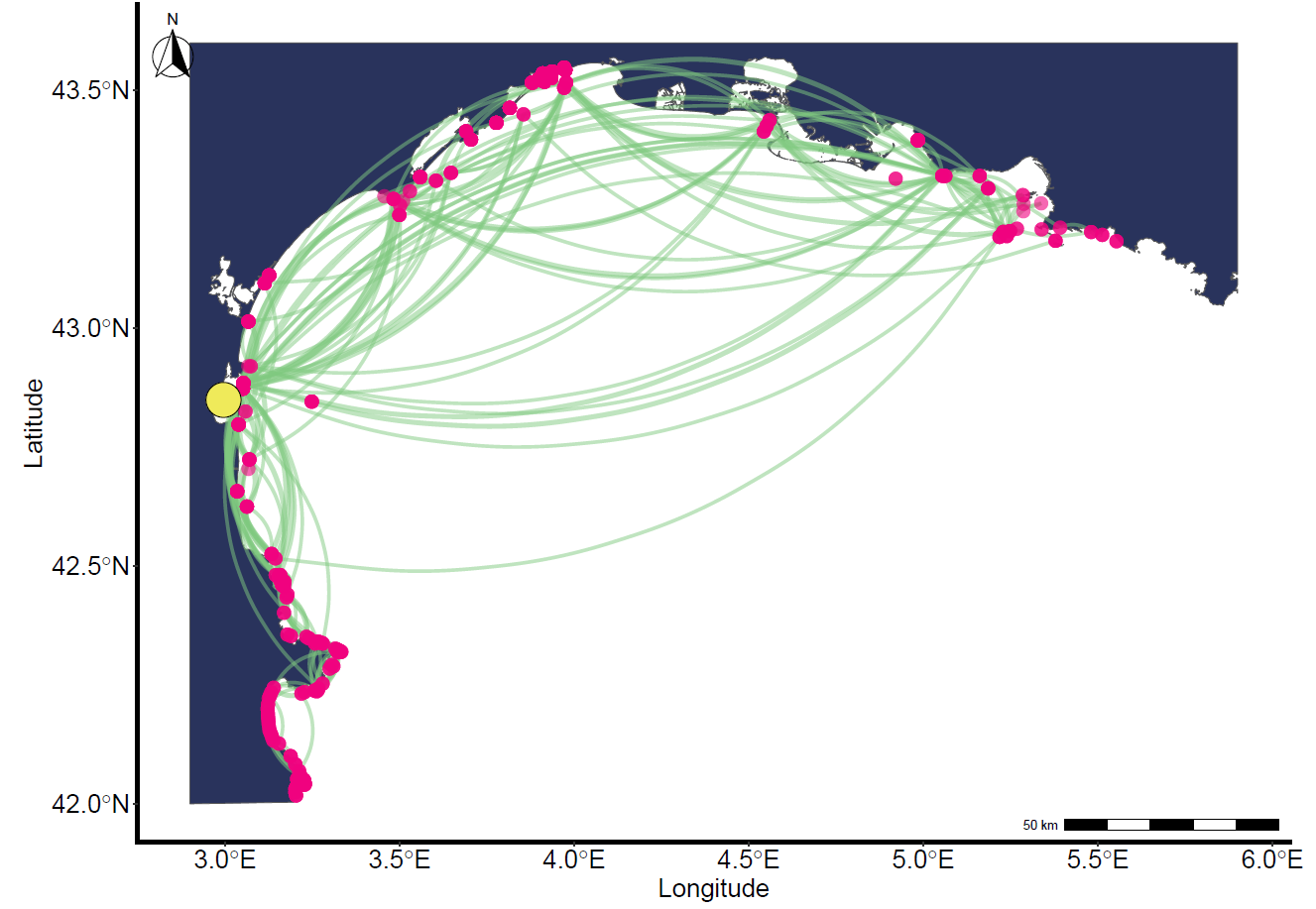


**Figure S5.** Aggregated regional spatial network from fish tagged in Leucate lagoon over the whole study period. Tagging location is shown in yellow, and stations are shown in pink.


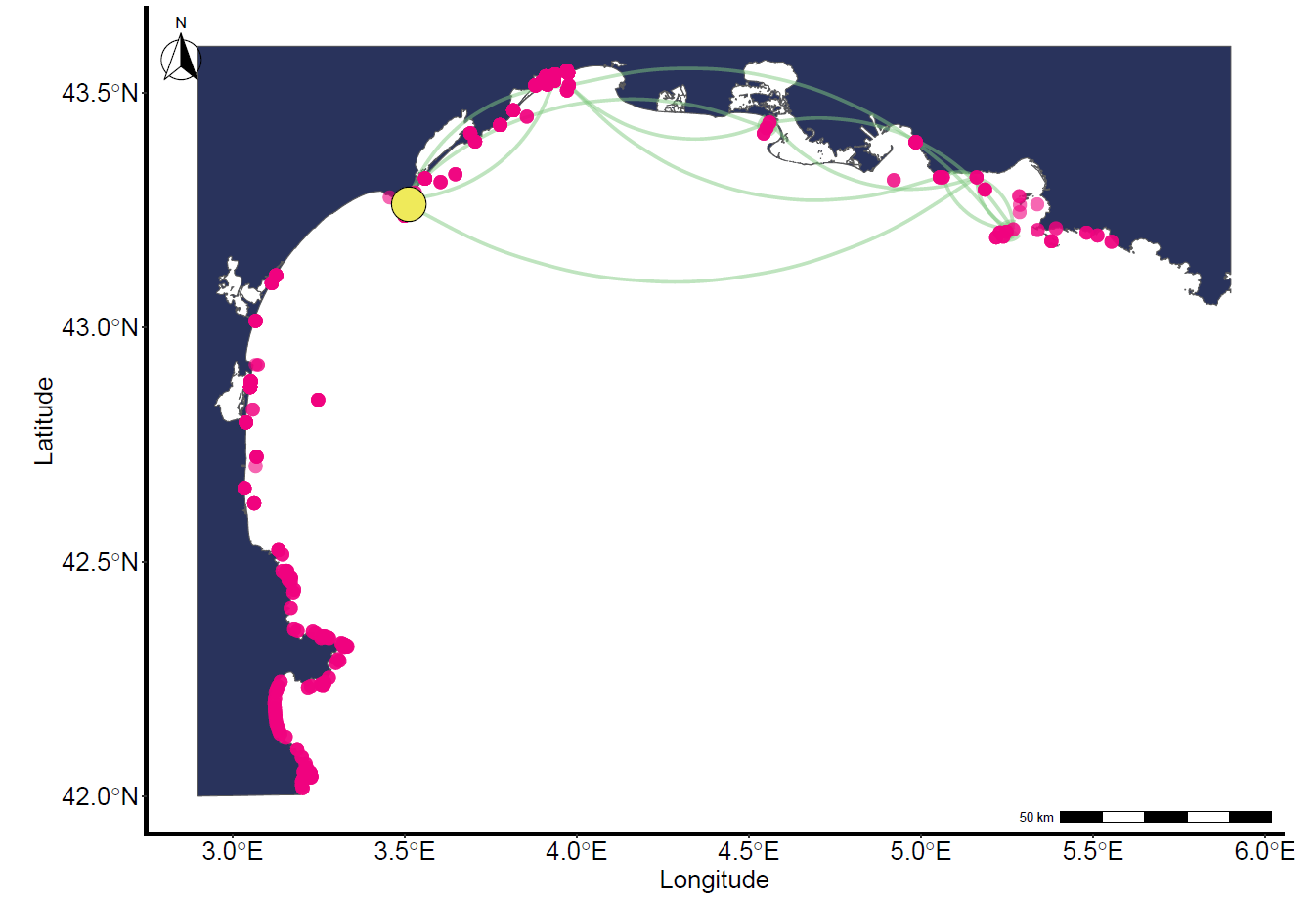


**Figure S6.** Aggregated regional spatial network from fish tagged in Thau sea over the whole study period. Tagging location is shown in yellow, and stations are shown in pink.


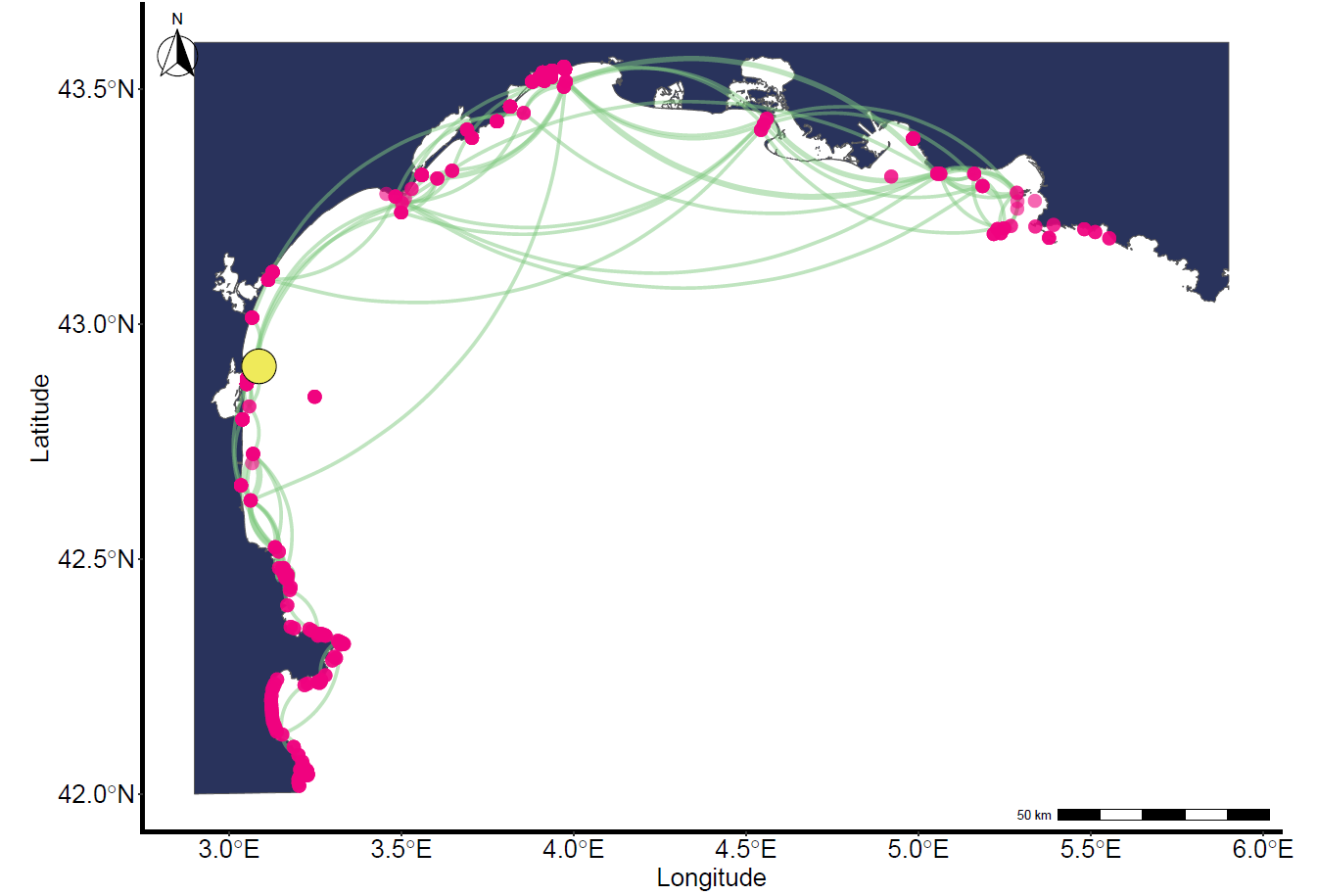


**Figure S7.** Aggregated regional spatial network from fish tagged in Leucate sea over the whole study period. Tagging location is shown in yellow, and stations are shown in pink.


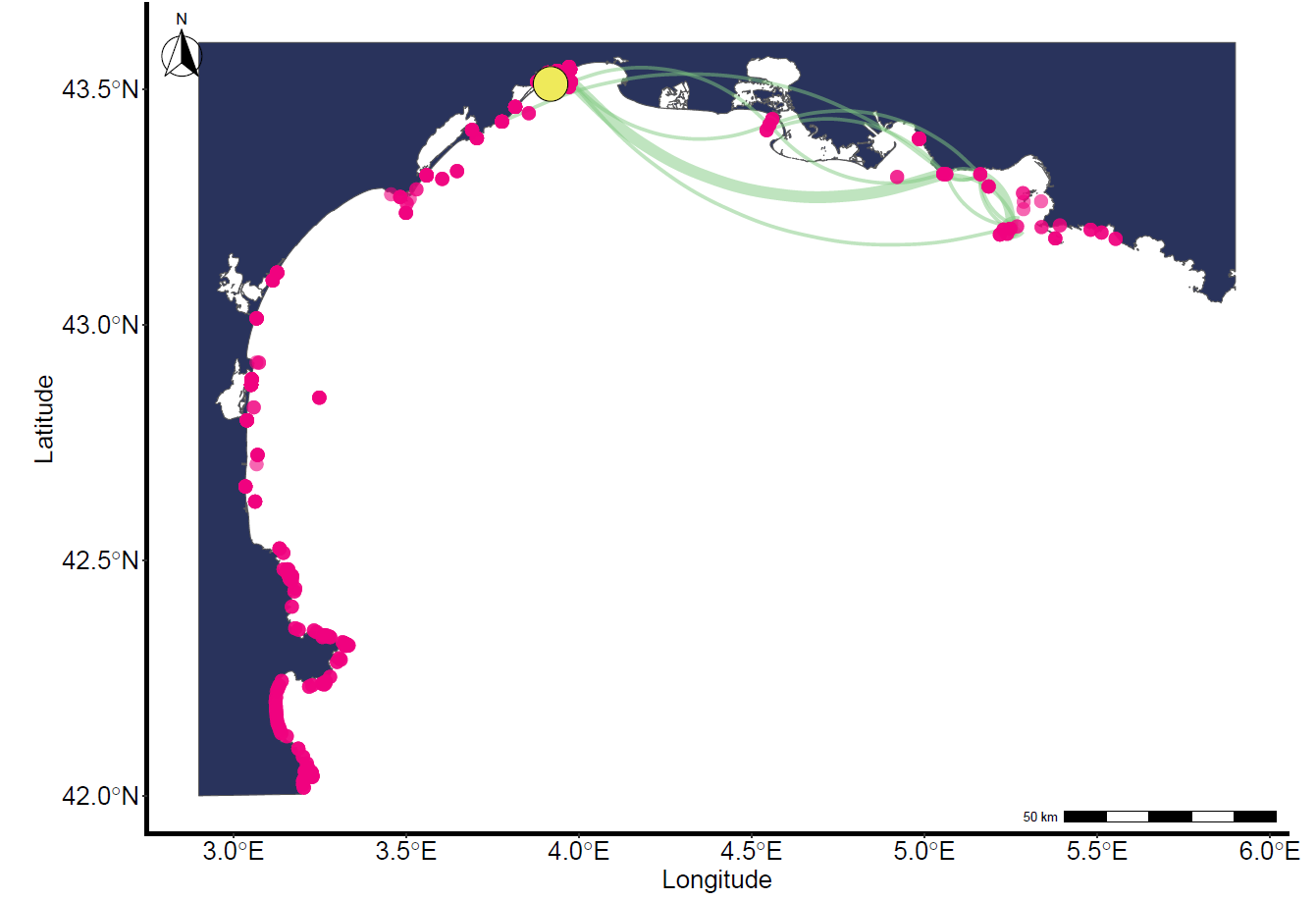


**Figure S8.** Aggregated regional spatial network from fish tagged in Vic sea over the whole study period. Tagging location is shown in yellow, and stations are shown in pink.


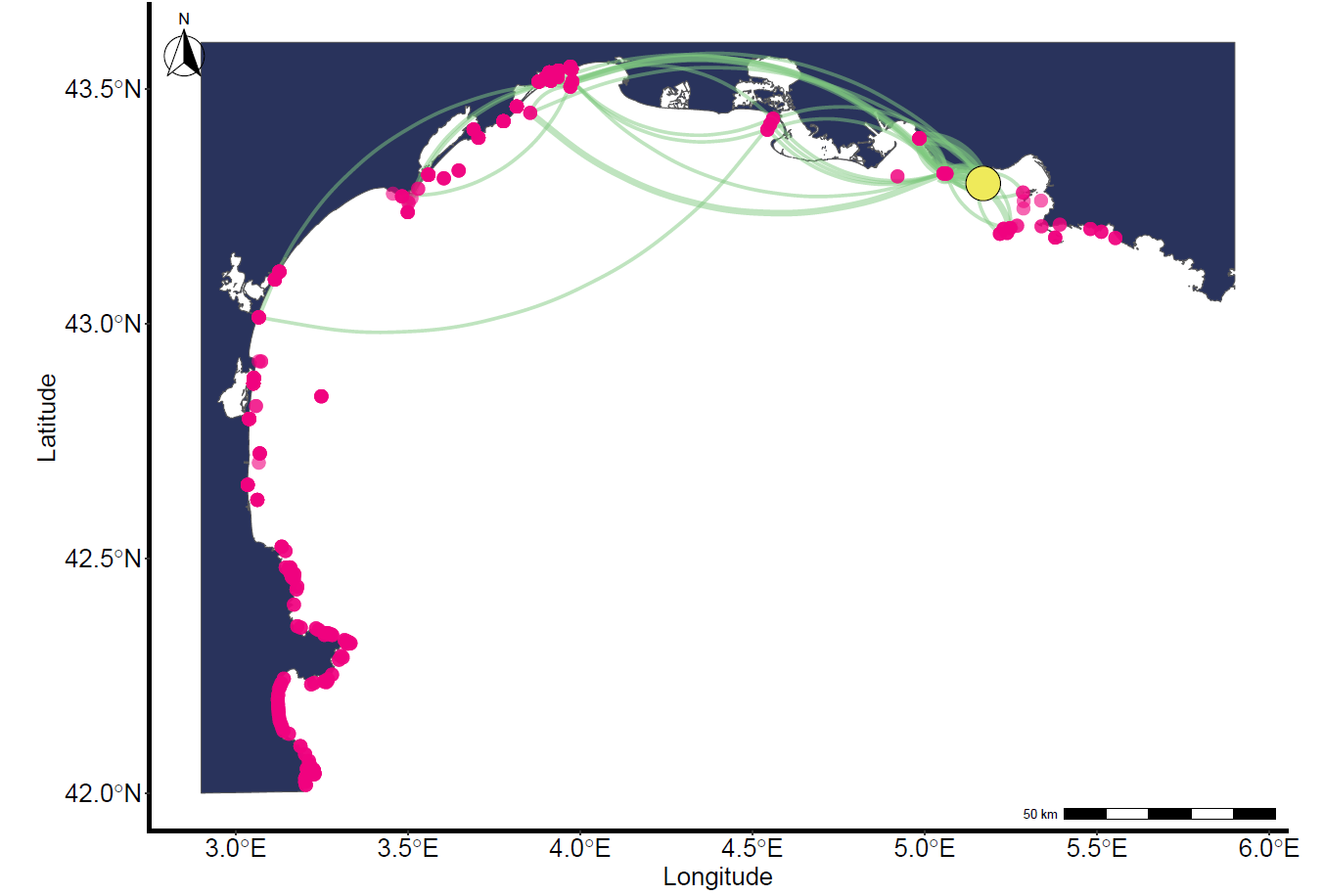


**Figure S9.** Aggregated regional spatial network from fish tagged in Marseille cote bleue sea over the whole study period. Tagging location is shown in yellow, and stations are shown in pink.


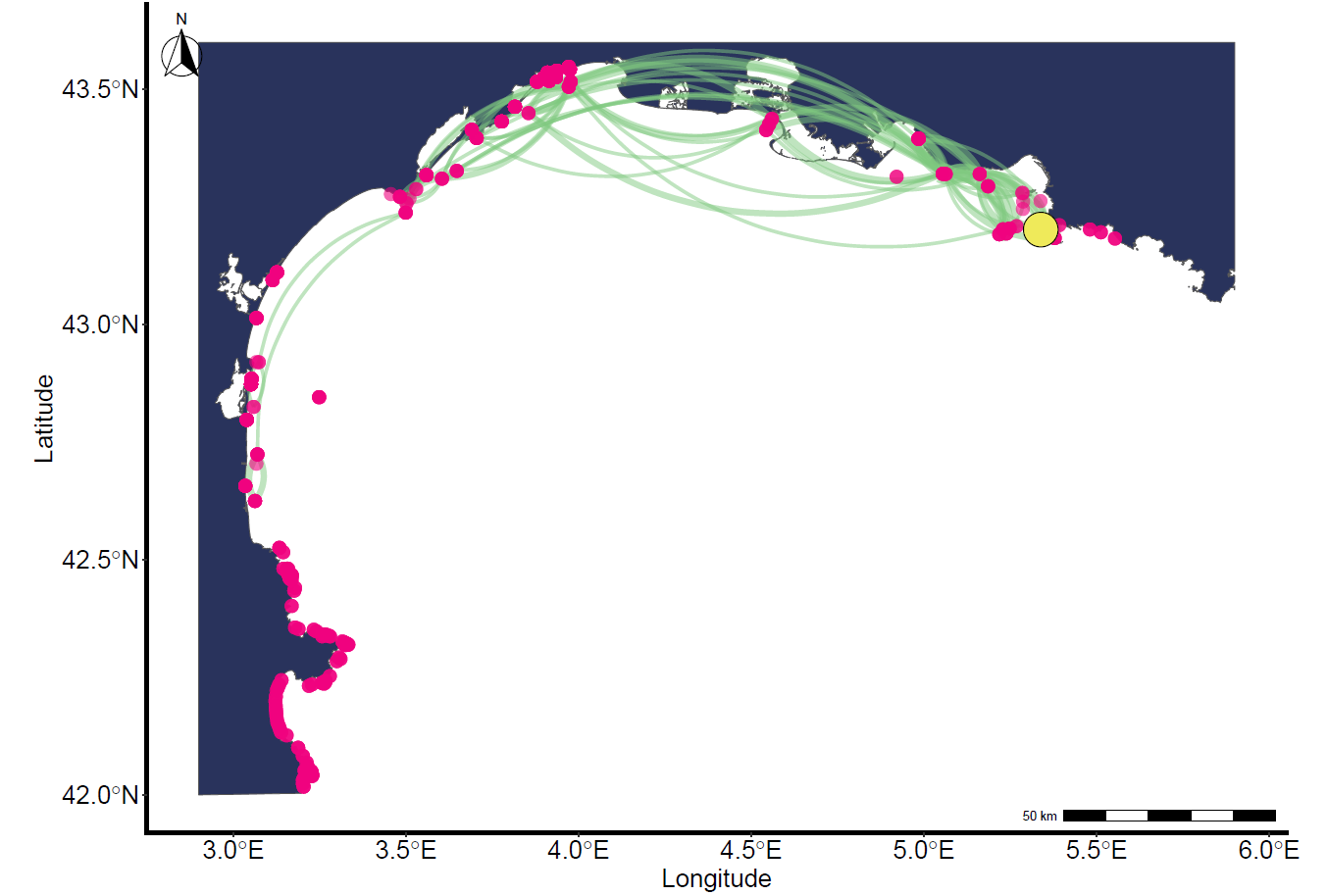


**Figure S10.** Aggregated regional spatial network from fish tagged in Marseille calanques sea over the whole study period. Tagging location is shown in yellow, and stations are shown in pink.


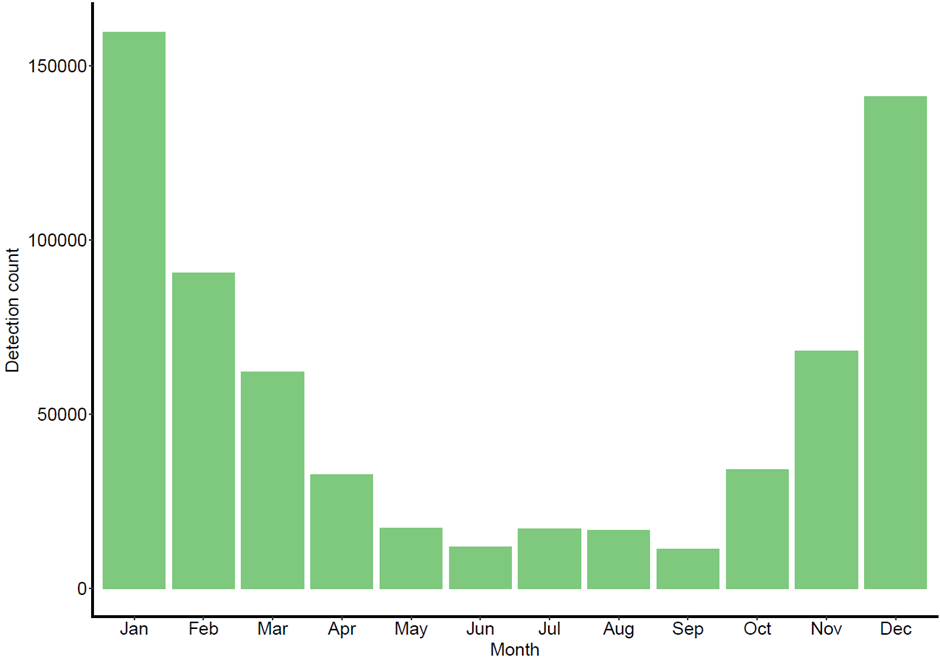


**Figure S11.** Sum of detections split by month during the duration of the study for at-sea stations.


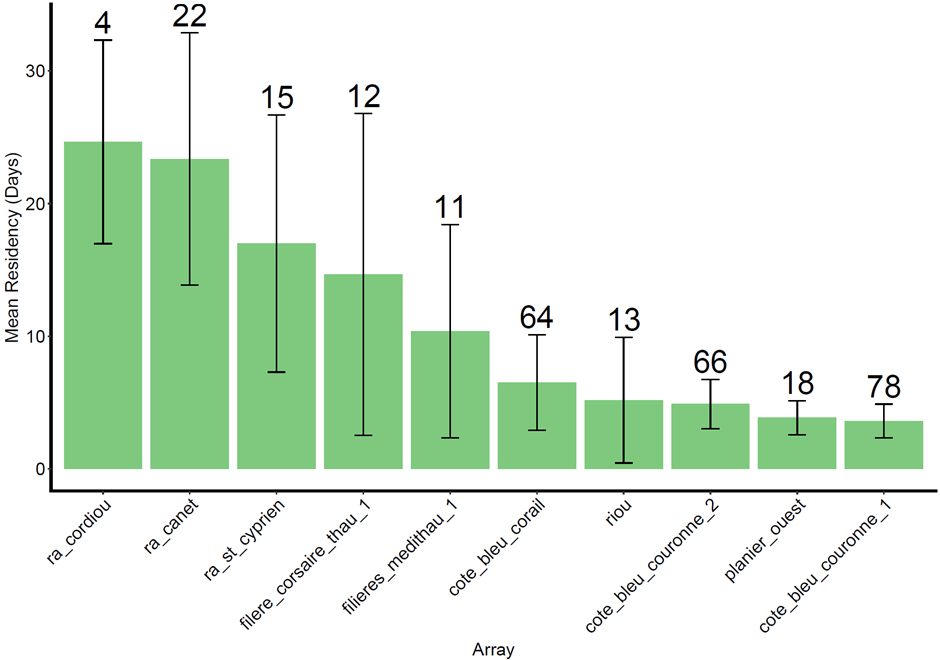


**Figure S12.** Mean residency time (days) +- SD for highest 10 sea stations in the CONNECT-MED project. Numbers above error bars are indicative of number of individuals recorded at array over the duration of the study.


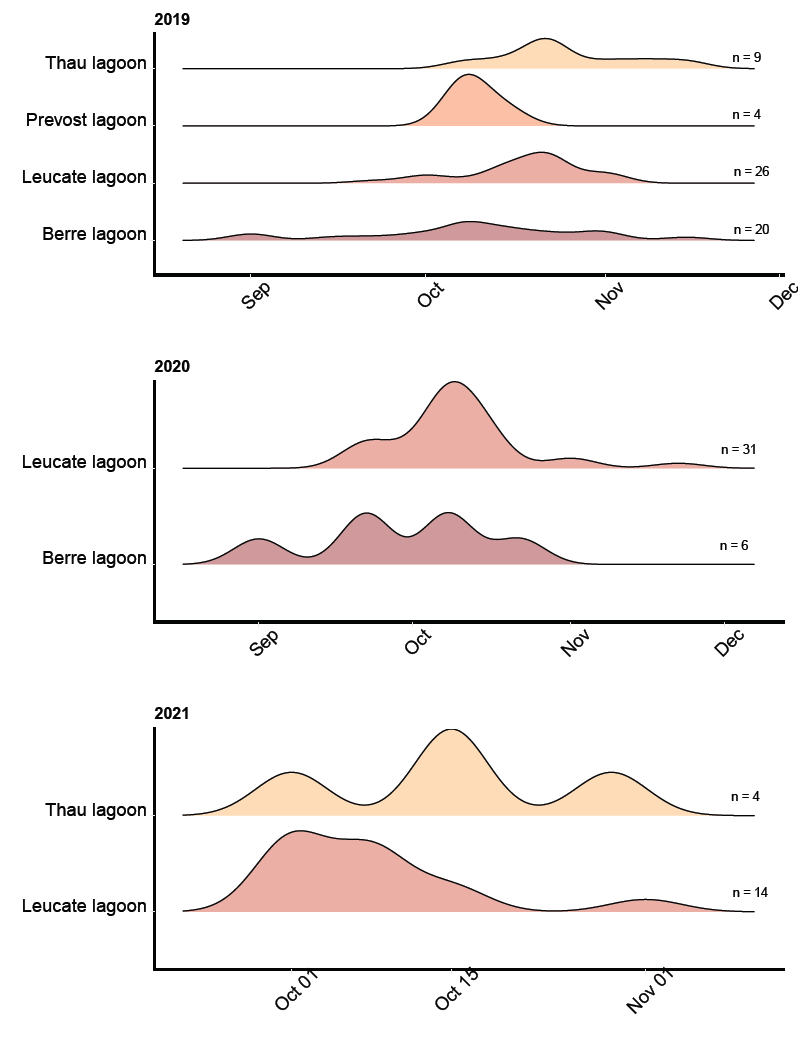


**Figure S13.** Density plots depicting the sum of individual last detections from their “home lagoons” aggregated into seven-day intervals. The ridges represent the distribution of detection events across different sites for 2019 (top), 2020 (middle) and 2021 (bottom). Only years in which more than one fish was detected are included.


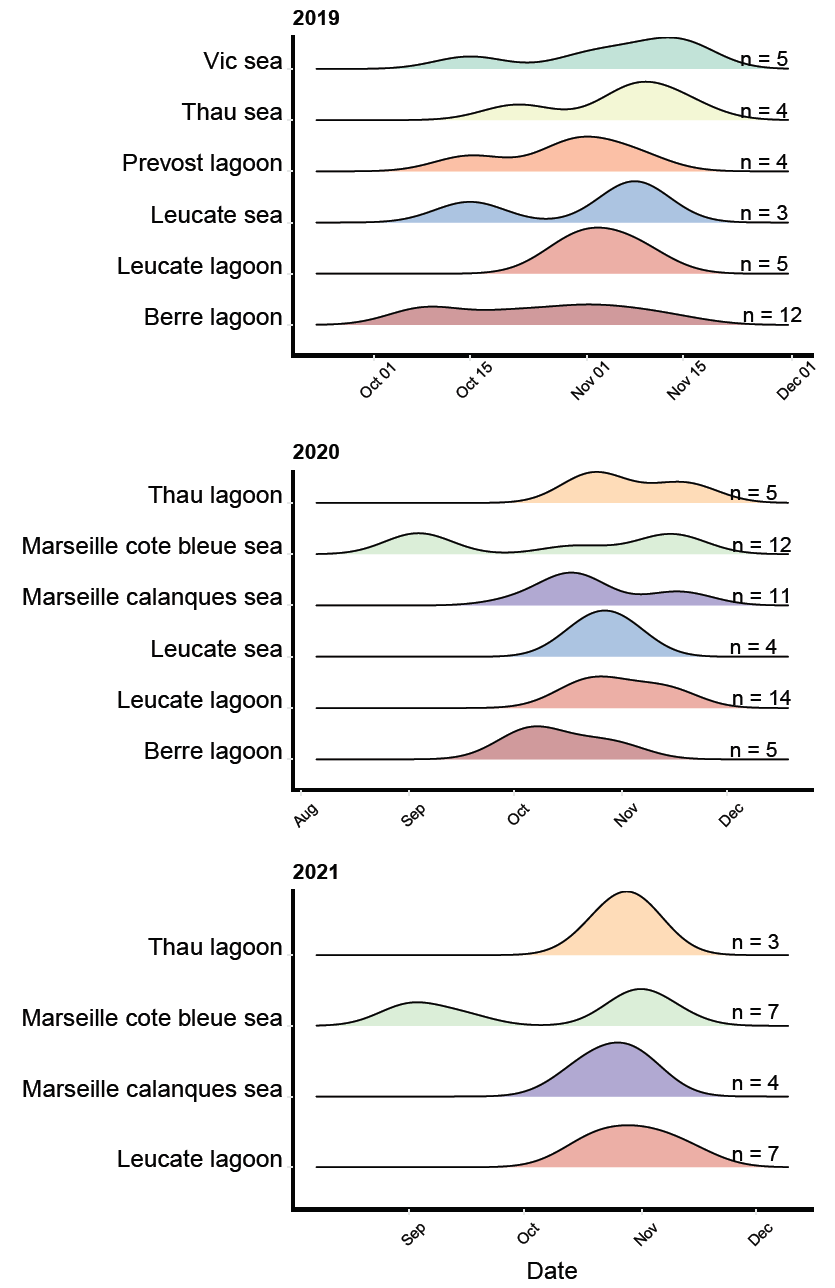


**Figure S14.** Density plots depicting the sum of individual first detections from each tagging location at spawning stations aggregated into seven-day intervals between September and November for each year of the study. The ridges represent the distribution of detection events across different sites for 2019 (top), 2020 (middle) and 2021 (bottom). Only years in which more than two fish were detected are included. For Marseille calanques sea and Marseille côte bleue sea only fish from previous tagging years are shown in each panel.


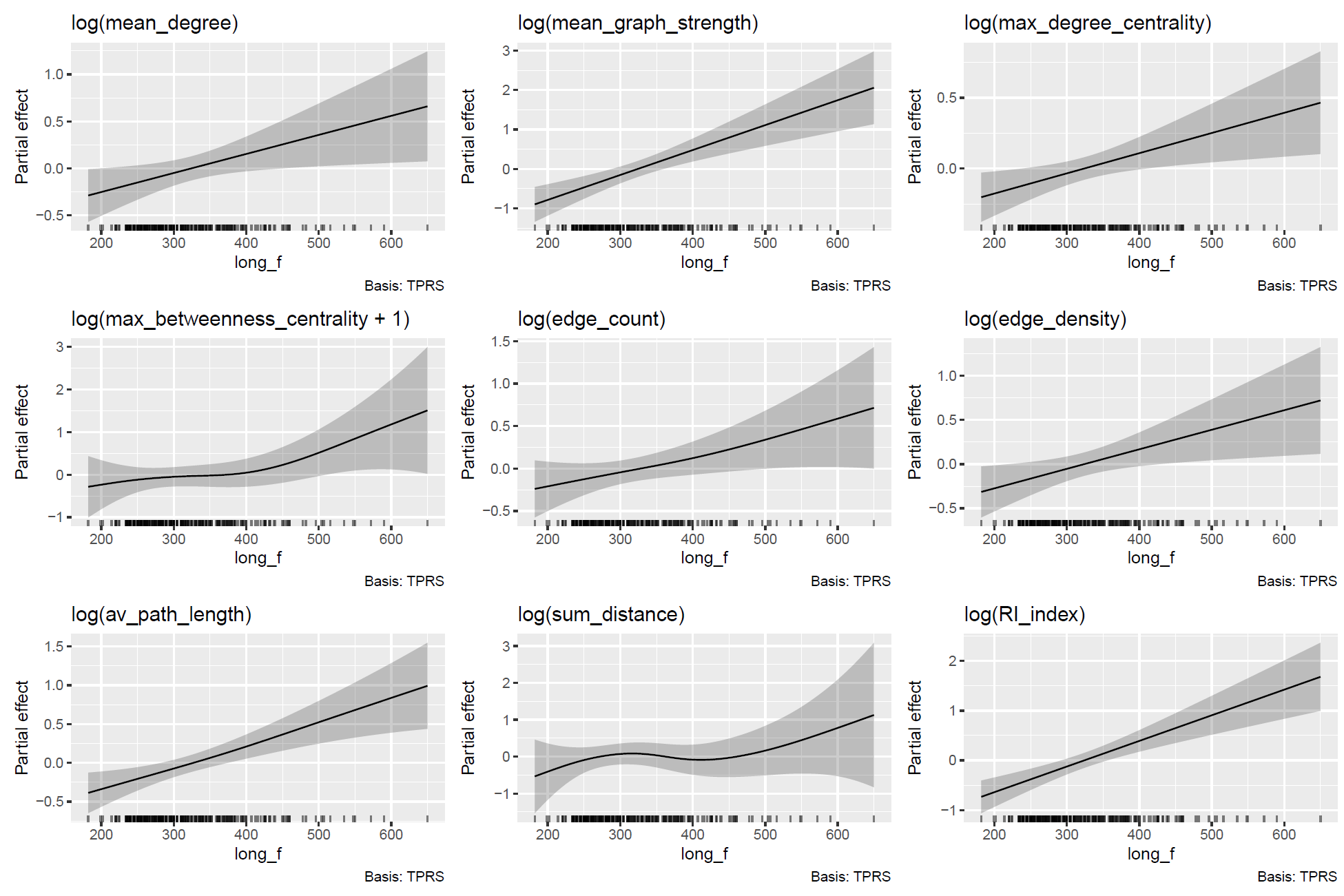


**Figure S15.** Partial effects of length (mm) on network and movement metrics. The y axes represent a partial effect on each of the response variables. Shaded areas represent the 95% confidence intervals around each smooth term. It should be noted that mgcv automatically defaults to a linear relationship in cases where smoothers are not required.

**
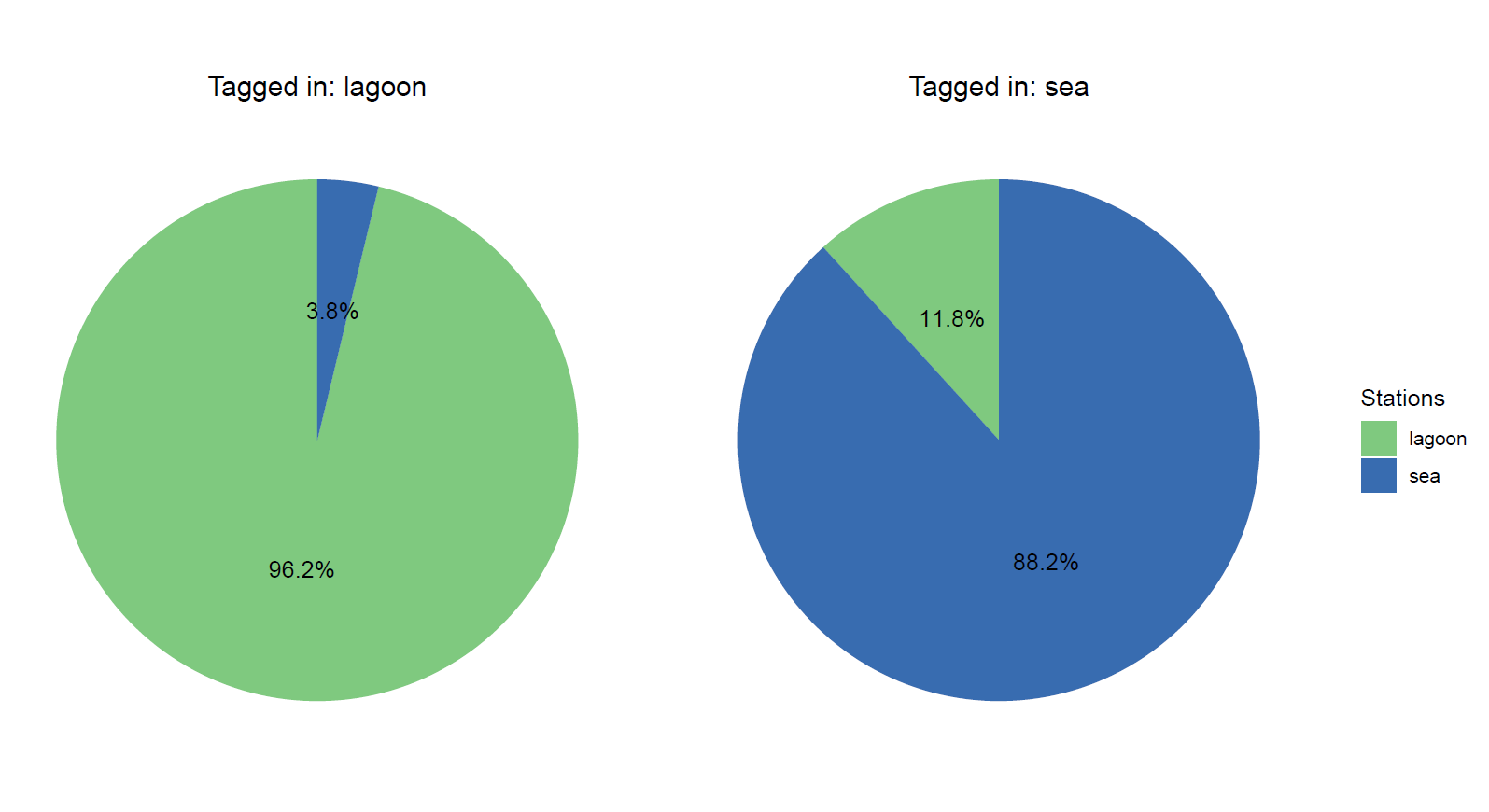
**

**Figure S16.** Percentage of fish detected at lagoon (n = 53) or sea stations (n = 17) during the foraging season as a function of where they were tagged (lagoons or sea). Note that only fish that visited one environment type are shown.

**
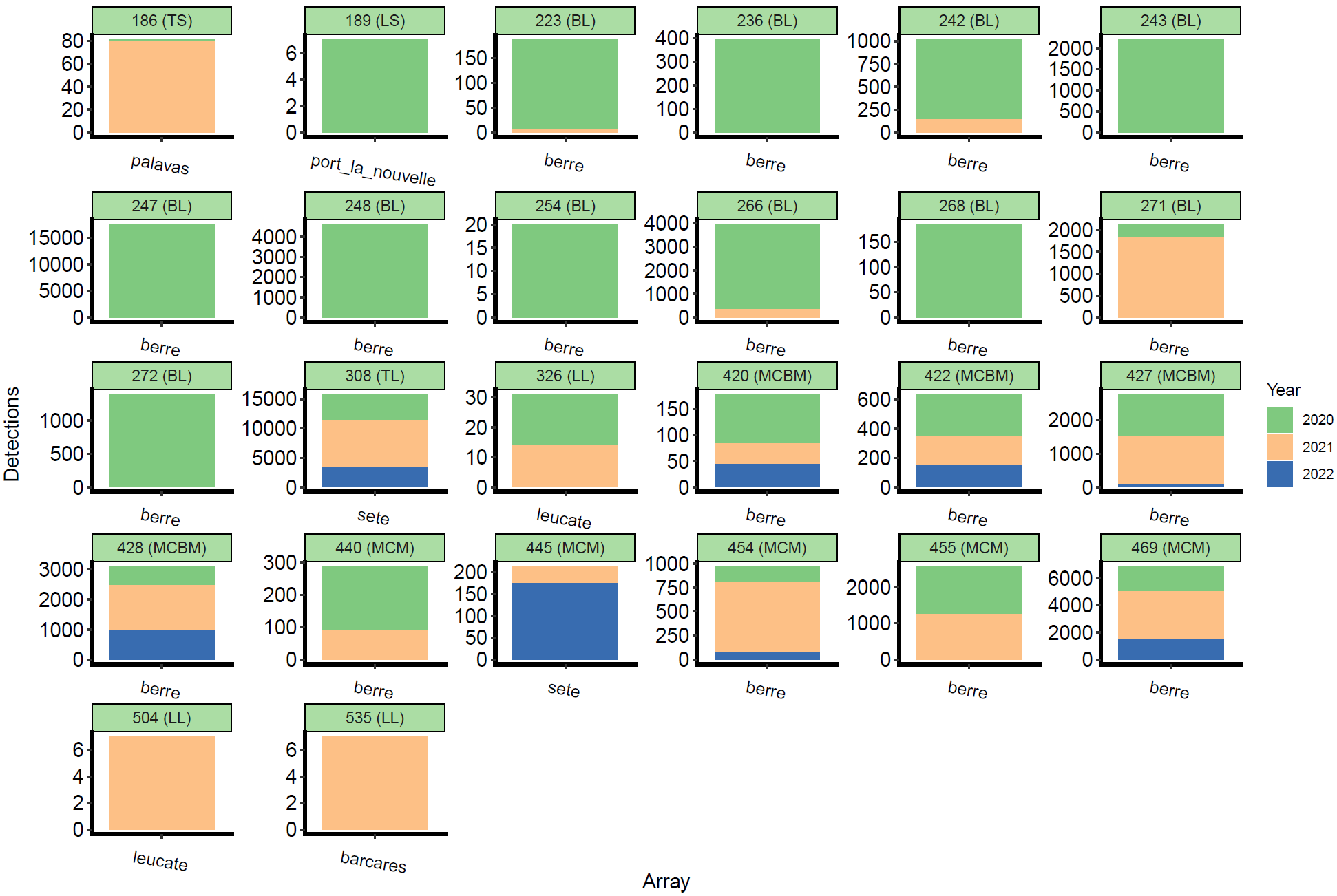
**

**Figure S17.** Detection counts at lagoon arrays during foraging for individuals that were detected multiple years (green = 2020, orange = 2021, and blue = 2022) at lagoons. Panel titles indicate tagging location (TS = Thau sea; LS = Leucate sea; BL = Berre Lagoon; TL= Thau Lagoon; LL= Leucate Lagoon; MCBM = Marseille cote bleue sea; MCM = Marseille calanques sea). Detections were restricted to the season following the release, thus detections from a fish's first foraging season are not shown.

**
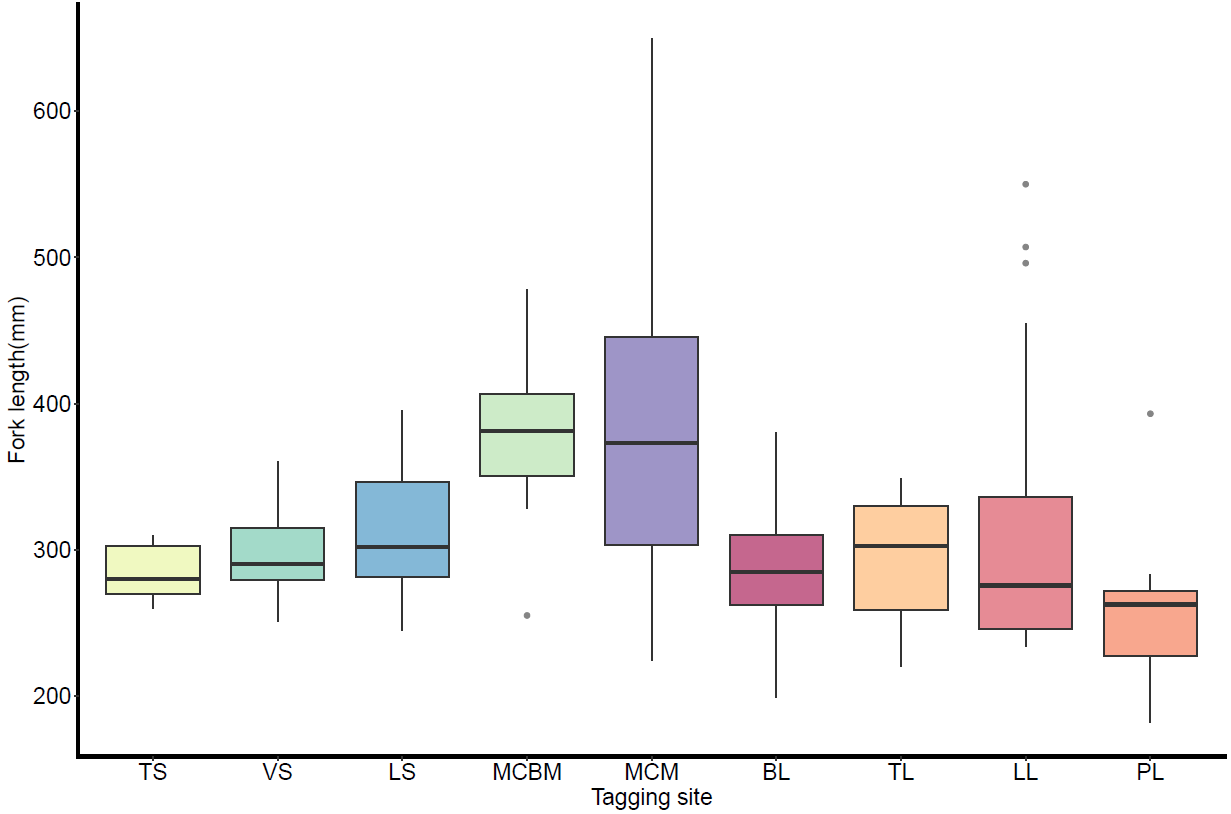
**

**Figure S18.** Size distributions of S.aurata tagged at each location (TS = Thau sea; VS = Vic Sea; LS = Leucate sea; MCBM = Marseille cote bleue sea; MCM = Marseille calanques sea; BL = Berre Lagoon; TL = Thau Lagoon; LL= Leucate Lagoon, and PL = Prevost Lagoon)

**Note S1.** The initial filtering the following fish were removed from analysis after visual inspection showed them displaying behaviours that were not coherent (e.g. very extensive periods at one station), if notes from tagging indicated potential issues, or other expert knowledge. This led to the removal of data associated to fish **#156**, and the partial removal of detections associated to **#198** and **#195**. Patterns of space use and residency suggested these tags were either shed or the fish had perished. Fish **#485** was removed from GAM results for being an extreme outlier. To identify individual fish please see data provided with script.
